# Amplitude Constrained MIMO Channels: Properties of Optimal Input Distributions and Bounds on the Capacity †

**DOI:** 10.3390/e21020200

**Published:** 2019-02-19

**Authors:** Alex Dytso, Mario Goldenbaum, H. Vincent Poor, Shlomo Shamai (Shitz)

**Affiliations:** 1 Department of Electrical Engineering, Princeton University, Princeton, NJ 08544, USA; 2 Department of Electrical Engineering, Technion-Israel Institute of Technology, Technion City, Haifa 32000, Israel

**Keywords:** MIMO, channel capacity, amplitude constraint, input distrbution, capacity bounds

## Abstract

In this work, the capacity of multiple-input multiple-output channels that are subject to constraints on the support of the input is studied. The paper consists of two parts. The first part focuses on the general structure of capacity-achieving input distributions. Known results are surveyed and several new results are provided. With regard to the latter, it is shown that the support of a capacity-achieving input distribution is a small set in both a topological and a measure theoretical sense. Moreover, explicit conditions on the channel input space and the channel matrix are found such that the support of a capacity-achieving input distribution is concentrated on the boundary of the input space only. The second part of this paper surveys known bounds on the capacity and provides several novel upper and lower bounds for channels with arbitrary constraints on the support of the channel input symbols. As an immediate practical application, the special case of multiple-input multiple-output channels with *amplitude constraints* is considered. The bounds are shown to be within a constant gap to the capacity if the channel matrix is invertible and are tight in the high amplitude regime for arbitrary channel matrices. Moreover, in the regime of high amplitudes, it is shown that the capacity scales linearly with the minimum between the number of transmit and receive antennas, similar to the case of average power-constrained inputs.

## 1. Introduction

While the capacity of a multiple-input multiple-output (MIMO) channel with an average power constraint is well understood [1], there is surprisingly little known about the capacity of the more practically relevant case in which the channel inputs are subject to *amplitude constraints*. Shannon was the first who considered a channel that is constrained in its amplitude [2]. In that paper, he derived corresponding upper and lower bounds and showed that in the low-amplitude regime, the capacity behaves as that of channel with an average power constraint. The next major contribution to this problem was a seminal paper of Smith [3] published in 1971. Smith showed that, for the single-input single-output (SISO) Gaussian noise channel with an amplitude-constrained input, the capacity-achieving inputs are discrete with finite support. In [4], this result is extended to peak-power-constrained quadrature Gaussian channels. Using the approach of Shamai [4], it is shown in [5] that the input distribution that achieves the capacity of a MIMO channel with an identity channel matrix and a Euclidian norm constraint on the input vector is discrete. Even though the optimal input distribution is known to be discrete, very little is known about the number or the optimal positions of the corresponding constellation points. To the best of our knowledge, the only case for which the input distribution is precisely known is considered in [6], where it is shown for the Gaussian SISO channel with an amplitude constraint that two point masses are optimal if amplitude values are smaller than 1.665 and three for amplitude values of up to 2.786. Finally, it has been shown very recently that the number of mass points in the support of the capacity-achieving input distribution of a SISO channel is of the order $O(A^{2})$ with *A* the amplitude constraint.

Based on a dual capacity expression, in [7], McKellips derived an upper bound on the capacity of a SISO channel that is subject to an amplitude constraint. The bound is asymptotically tight; that is, for amplitude values that tend to infinity. By cleverly choosing an auxiliary channel output distribution in the dual capacity expression, the authors of [8] sharpened McKellips’ upper bound and extended it to parallel MIMO channels with a Euclidian norm constraint on the input. The SISO version of the upper bound in [8] has been further sharpened in [9] by yet another choice of auxiliary output distribution. In [10], asymptotic lower and upper bounds for a 2×2 MIMO channel are presented and the gap between the bounds is specified.

In this work, we make progress on this open problem by deriving several new upper and lower bounds that hold for channels with *arbitrary constraints* on the support of the channel input distribution and then apply them to the practically relevant special case of MIMO channels that are subject to amplitude-constraints.

### 1.1. Contributions and Paper Organization

The remainder of the paper is organized as follows. The problem is stated in Section 2. In Section 3, we study properties of input distributions that achieve the capacity of input-constrained MIMO channels. The section reviews known results on the structure of optimal input distributions and presents several new results. In particular, Theorem 3 shows that the support of a capacity-achieving input distribution must necessarily be a small set both topologically and measure theoretically. Moreover, Theorem 8 characterizes conditions on the channel input space as well as on the channel matrix such that the support of the optimal input distribution is concentrated on the boundary of the channel input space.

In Section 4, we derive novel upper and lower bounds on the capacity of a MIMO channel that is subject to an arbitrary constraint on the support of the input. In particular, three families of upper bounds are proposed, which are based on: (i) the maximum entropy principle (see the bound in Theorem 9); (ii) the dual capacity characterization (see the bound in Theorem 10); and (iii) a relationship between mutual information and the minimum mean square error that is known as the I-MMSE relationship (see the bound in Theorem 11). On the other hand, Section 4 provides three different lower bounds. The first one is given in Theorem 12 and is based on the entropy power inequality. The second one (see Theorem 13) is based on a generalization of the celebrated Ozarow–Wyner bound [11] to the MIMO case. The third upper bound (see Theorem 14) is based on Jensen’s inequality and depends on the characteristic function of the channel input distribution.

In Section 5, we evaluate the performance of our bounds by studying MIMO channels with invertible channel matrices. In particular, Theorem 17 states that our upper and lower bounds are within $n\log_{2}\left(\rho \right)$ bits, where ρ is the packing efficiency and *n* the number of transmit and receive antennas. For diagonal channel matrices, it is then shown (see Theorem 18) that the Cartesian product of simple pulse-amplitude modulation (PAM) constellations achieves the capacity to within 1.64n bits.

Section 6 is devoted to MIMO channels with arbitrary channel matrices. It is shown that, in the regime of high amplitudes, similar to the case of average power-constrained channel inputs, the capacity scales linearly with the minimum of the number of transmit and receive antennas.

In Section 7, our upper and lower bounds are applied to the SISO case, which are then compared with bounds known from the literature. Finally, Section 8 concludes the paper. Note that parts of the results in this paper were also published in [12].

### 1.2. Notation

Vectors are denoted as bold lowercase letters, random vectors as bold uppercase letters, and matrices as bold uppercase sans serif letters (e.g., x, X, X). For any deterministic vector $x\in R^{n}$, n∈N, we denote the Euclidian norm of x by ∥x∥. For some random $X\in \mathrm{supp}\left(X\right)\subseteq R^{n}$ and any p>0, we define

(1) $${∥X∥}_{p}:={\left(\frac{1}{n}{E[∥X∥}^{p}]\right)}^{\frac{1}{p}},$$

where supp(X) denotes the support of X. Note that for p≥1, the quantity in Equation (1) defines a norm and for n=1 we simply have ${∥X∥}_{p}^{p}={E[|X|}^{p}]$.

The norm of a matrix $H\in R^{n\times n}$ is defined as

$$∥H∥:=\underset{x:x\neq 0}{\sup}\frac{∥Hx∥}{∥x∥},$$

whereas Tr(H) is denoting its trace. The n×n identity matrix is represented as $I_{n}$.

Let S be a subset of $R^{n}$. Then,

$$\mathrm{Vol}\left(S\right):=\int_{S}\;dx$$

denotes its volume. Moreover, the boundary of S is denoted as ∂S.

Let $R_{+}:=\left\{x\in R:x\geq 0\right\}$. We define an *n*-dimensional ball or radius $r\in R_{+}$ centered at $x\in R^{n}$ as the set

$$B_{x}\left(r\right):=\{y\in R^{n}:∥x-y∥\leq r\}.$$

Recall that, for any $x\in R^{n}$ and $r\in R_{+}$,

$$\mathrm{Vol}\left(B_{x}\left(r\right)\right)=\frac{\pi^{\frac{n}{2}}}{\Gamma \left(\frac{n}{2}+1\right)}r^{n},$$

where Γ(z) denotes the gamma function.

For any matrix $H\in R^{k\times n}$ and some $S\subset R^{n}$, we define

$$HS:=\{y\in R^{k}:y=Hx\;,\;x\in S\}.$$

Note that for an invertible $H\in R^{n\times n}$, we have

Vol(HS)=|det(H)|Vol(S)

with det(H) the determinant of H. We define the maximum and minimum radius of a set $S\subset R^{n}$ that contains the origin as

$$\begin{array}{cc} r_{\max}\left(S\right) & :=\min\{r\in R_{+}:S\subset B_{0}\left(r\right)\},\\ r_{\min}\left(S\right) & :=\max\{r\in R_{+}:B_{0}\left(r\right)\subseteq S\}. \end{array}$$

For a given vector $a=\left(a_{1},\cdots ,a_{n}\right)\in R_{+}^{n}$, we define

$$\mathrm{Box}\left(a\right):=\{x\in R^{n}:|x_{i}|\leq a_{i},i=1,\cdots ,n\}$$

and the smallest box containing a given set $S\subset R^{n}$ as

Box(S):=inf{Box(a):S⊆Box(a)},

respectively.

The entropy of any discrete random object X is denoted as H(X), whereas h(X) (i.e., the differential entropy) is used whenever X is continuous. The mutual information between two random objects X and Y is denoted as I(X;Y) and N(m,C) denotes the multivariate normal distribution with mean vector m and covariance matrix C. Finally, $\log_{a}^{+}\left(x\right):=\max\left\{\log_{a}\left(x\right),0\right\}$, for any base a>0, Q(x), x∈R, denotes the Q-function, and $\delta_{x}\left(y\right)$ the Kronecker delta, which is one for x=y and zero otherwise.

## 2. Problem Statement

Consider a MIMO system with $n_{t}\in N$ transmit and $n_{r}\in N$ receive antennas. The corresponding $n_{r}$-dimensional channel output for a single channel use is of the form

Y=HX+Z,

for some fixed channel matrix $H\in R^{n_{r}\times n_{t}}$ (considering a real-valued channel model is without loss of generality). Here and hereafter, we assume $Z∼N(0,I_{n_{r}})$ is independent of the channel input $X\in R^{n_{t}}$ and H is known to both the transmitter and the receiver.

Now, let $X\subset R^{n_{t}}$ be a *convex and compact* channel input space that contains the origin (i.e., the length-$n_{t}$ zero vector) and let $F_{X}$ denote the cumulative distribution function of X. As of the writing of this paper, the capacity

(2) $$C\left(X,H\right):=\max_{F_{X}:X\in X}I\left(X;Y\right)=\max_{F_{X}:X\in X}I\left(X;HX+Z\right),$$

of this channel is unknown and we are interested in finding novel lower and upper bounds. Even though most of the results in this paper hold for arbitrary convex and compact X, we are mainly interested in the two important special cases:

(i) per-antenna amplitude constraints, i.e., X=Box(a) for some given $a=\left(A_{1},\cdots ,A_{n_{t}}\right)\in R_{+}^{n_{t}}$; and
(ii) $n_{t}$-dimensional amplitude constraint, i.e., $X=B_{0}\left(A\right)$ for some given $A\in R_{+}$.

**Remark** **1.** *Note that determining the capacity of a MIMO channel with average per-antenna power constraints is also still an open problem and has been solved for some special cases only [13,14,15,16,17].*

## 3. Properties of an Optimal Input Distribution

Unlike the special cases of real and complex-valued SISO channels (i.e., $n_{t}=n_{r}=1$), the structure of the capacity-achieving input distribution, denoted as $F_{X}^{⋆}$, is in general not known. To motivate why in this paper we are seeking for novel upper and lower bounds on the capacity (Equation (2)) instead of trying to solve the optimization problem directly, in this section we first summarize properties optimal input distributions must posses, which demonstrate how complicated the optimization problem actually is. Note that, whereas an optimal input distribution always exists, it does not necessarily need to be unique.

### 3.1. Necessary and Sufficient Conditions for Optimality

To study properties of an optimal input distribution, we need the notion of a point of increase of probability distribution.

**Definition** **1.** *(Points of Increase of a Distribution) A point $x\in R^{n}$, n∈N, is said to be a point of increase of a given probability distribution $F_{X}$ if for any open set $A\subset R^{n}$ containing x, $F_{X}\left(A\right)>0$.*

The following result provides necessary and sufficient conditions for the optimality of a channel input distribution.

**Theorem** **1.** *Let $F_{X}$ be some given channel input distribution and let $E(F_{X})\subset X$ denote the set of points of increase of $F_{X}$. Then, the following holds:*

- *$F_{X}$ is capacity-achieving if and only if the Karush–Kuhn–Tucker (KKT) conditions*
  (3a) $$\begin{array}{cc} h(x;F_{X}) & \leq h(HX+Z),\;x\in X, \end{array}$$
  (3b) $$\begin{array}{cc} h(x;F_{X}) & =h\left(HX+Z\right),\;x\in E\left(F_{X}\right)\subset X, \end{array}$$
  *are satisfied [3,18], where*
  $$\begin{array}{c} h\left(x;F_{X}\right):=-\int_{R^{n_{r}}}\frac{1}{{\left(2\pi \right)}^{\frac{n_{r}}{2}}}e^{-\frac{{∥y-Hx∥}^{2}}{2}}\log_{2}\left(f_{Y}\left(y\right)\right)dy \end{array}$$
  *with $f_{Y}$ being the probability density of the channel output induced by the channel input $X∼F_{X}$.*
- *$F_{X}$ is unique and symmetric if H is left invertible [18].*
- *$F_{Y}$ (i.e., the channel output distribution) is unique [18,19].*

### 3.2. General Structure of Capacity-Achieving Input Distributions

Theorem 1 can be used to find general properties of the support of a capacity-achieving input distribution, which we will do in this subsection.

**Remark** **2.** *Fully characterizing an input distribution that achieves the capacity of a general MIMO channel with per-antenna or an $n_{t}$-dimensional amplitude constraint is still an open problem. To the best of our knowledge, the only general available for showing that discrete channel inputs are optimal was developed by Smith in [3] for the amplitude and variance-constrained Gaussian SISO channel. Since then, it has been useful to also characterize the optimal input distribution of several other SISO channels (see, for instance, [4,20,21,22,23,24]). The method relies on the following series of steps:*

1. *Towards a contradiction, it is assumed that the set of points of increase $E(F_{X})$ is infinite.*
2. *The assumption in Step 1 is then used to establish a certain property of the function $h(x;F_{X})$ on the input space X. For example, by showing that $h(x;F_{X})$ has an analytic continuation to C. Then, by means of the Identity Theorem of complex analysis and the Bolzano–Weierstrass Theorem [25], Smith was able to show that $h(x;F_{X})$ must be constant.*
3. *By using either the Fourier or Laplace transform of $h(x;F_{X})$ together with the property of $h(x;F_{X})$ established in Step 2, a new a property of the channel output distribution $F_{Y}$ is established. For example, Smith was able to show that $F_{Y}$ must be constant.*
4. *A conclusion out of Step 3 is used to reach a contradiction. The contradiction implies that $E(F_{X})$ must be finite. For example, to reach a contradiction, Smith was using the fact that the channel output distribution $F_{Y}$ results from a convolution with a Gaussian probability density, which cannot be constant.*

**Remark** **3.** *Under the restriction that the output space, Y, of a Gaussian SISO channel is finite and the channel input space, X, is subject to an amplitude constraint, Witsenhausen has shown in [26] that the capacity-achieving input distribution is discrete with the number of mass points bounded as |X|≤|Y|. The approach of Witsenhausen, however, does not use the variational technique of Smith and relies on arguments from convex analysis instead.*

According to Remark 2, assuming in the MIMO case that $E(F_{X})$ is of infinite cardinality does not help (or at least it is not clear how this assumption should be used) in showing that the capacity-achieving input distribution is discrete and finite. However, by using the weaker assumption that $E(F_{X})$ contains a non-empty open subset in conjunction with the following version of the Identity Theorem, we can show that the support of the optimal input distribution is a small set in a certain topological sense.

**Theorem** **2.** (Identity Theorem for Real-Analytic Functions [27]) *For some n∈N let U be a subset of $R^{n}$ and f,g:U→R be two real-analytic functions that agree on a set A⊆U. Then, f and g agree on $R^{n}$ if one of the following two conditions is satisfied:*

(i) A is an open set.
(ii) A is a set of positive Lebesgue measure.

*Furthermore, for n=1, it suffices for A to be an arbitrary set with an accumulation point.*

We also need the definitions of a dense and a nowhere dense set.

**Definition** **2.** *(Dense and Nowhere Dense Sets) A subset A⊂X is said to be* dense *in the set X if every element x∈X either belongs to A or is an accumulation point of A. A subset A⊂X is said to be* nowhere dense *if for every nonempty open subset U⊂X, the intersection A∩U is not dense in U.*

With Theorem 2 at our disposal, we are now able to prove the following result on the structure of the support of the optimal input distribution.

**Theorem** **3.** *The set of points of increase $E(F_{X}^{⋆})$ of an optimal input distribution $F_{X}^{⋆}$ is a nowhere dense subset of X that is of Lebesgue measure zero.*

**Proof.**  It is not difficult to show that $h(x;F_{X}^{⋆})$ is a real-analytic function on $R^{n_{t}}$ ([18] Proposition 5). Now, in order to prove the result, we follow a series of steps similar to those outlined in Remark 2. Towards a contradiction, assume that the set of points of increase $E(F_{X}^{⋆})$ of $F_{X}^{⋆}$ is *not* a nowhere dense subset of X. Then, according to Definition 2, there exists an open set U⊂X such that $E(F_{X}^{⋆})\cap U$ is dense in U.

By using the KKT condition in Equation (3b), we have that $h(x;F_{X}^{⋆})$ is constant on the intersection $E(F_{X}^{⋆})\cap U$. Thus, as $E(F_{X}^{⋆})\cap U$ is dense in U, it follows by the properties of continuous functions (real-analytic functions are continuous) that $h(x;F_{X}^{⋆})$ is also constant on U. Moreover, as U is an open set, Theorem 2 implies that $h(x;F_{X}^{⋆})$ must also be constant on $R^{n_{t}}$. This, however, leads to a contradiction as $h(x;F_{X}^{⋆})$ cannot be constant on all of $R^{n_{t}}$, which can be shown by taking the Fourier transform of $h(x;F_{X}^{⋆})$ and solving for the probability density $f_{Y}\left(y\right)$ of the channel output (the reader is referred to [3] for details). Therefore, we conclude that $E(F_{X}^{⋆})$ is a nowhere dense subset of X.

Showing that $E(F_{X}^{⋆})$ has Lebesgue measure zero follows along similar lines by assuming that $E(F_{X}^{⋆})$ is a set of positive measure. Then, Property (ii) of Theorem 2 can be used to conclude that $h(x;F_{X}^{⋆})$ must be zero on all of U. This again leads to a contradiction, which implies that $E(F_{X}^{⋆})$ must be of Lebesgue measure zero. □

**Remark** **4.** *Note that if $X=B_{0}\left(A\right)$ for some $A\in R_{+}$ and $h(x;F_{X}^{⋆})$ is* orthogonally equivariant *(i.e., it only depends on ∥x∥), then $E(F_{X}^{⋆})$ can be written as a union of concentric spheres. That is,*

(4) $$E\left(F_{X}^{⋆}\right)=\underset{j}{⋃}C\left(A_{j}\right)$$

*with $C\left(A_{j}\right):=\{x\in R^{n_{t}}:∥x∥=A_{j}\}$ for some $A_{j}\in R_{+}$. To see this, let*

$$g\left(x\right):=h\left(x;F_{X}^{⋆}\right)-h\left(HX+Z\right)$$

*and observe that if $x\in E(F_{X}^{⋆})$, then g(x)=0. Combining this with the symmetry of the function ∥x∥↦g(∥x∥), we have that (We know that it is abuse of notation to use the same letter for the functions x↦g(x) and ∥x∥↦g(∥x∥) even if they are different. It is an attempt to say in a compact way that g is orthogonally equivariant.)*

$$\forall ∥x∥=∥y∥:x\in E\left(F_{X}^{⋆}\right)\Rightarrow y\in E\left(F_{X}^{⋆}\right).$$

*Moreover, this implies that*

$$E\left(F_{X}^{⋆}\right)=\underset{j\in I}{⋃}C\left(A_{j}\right),$$

*where I is possibly of infinite cardinality. In fact, I has finite cardinality. To see this, note that, if g(x) is real-analytic, then so is g(∥x∥). However, as ∥x∥↦g(∥x∥) is a non-zero real-analytic function on R, it can have at most finitely many zeros on an interval.*

**Figure 1:**
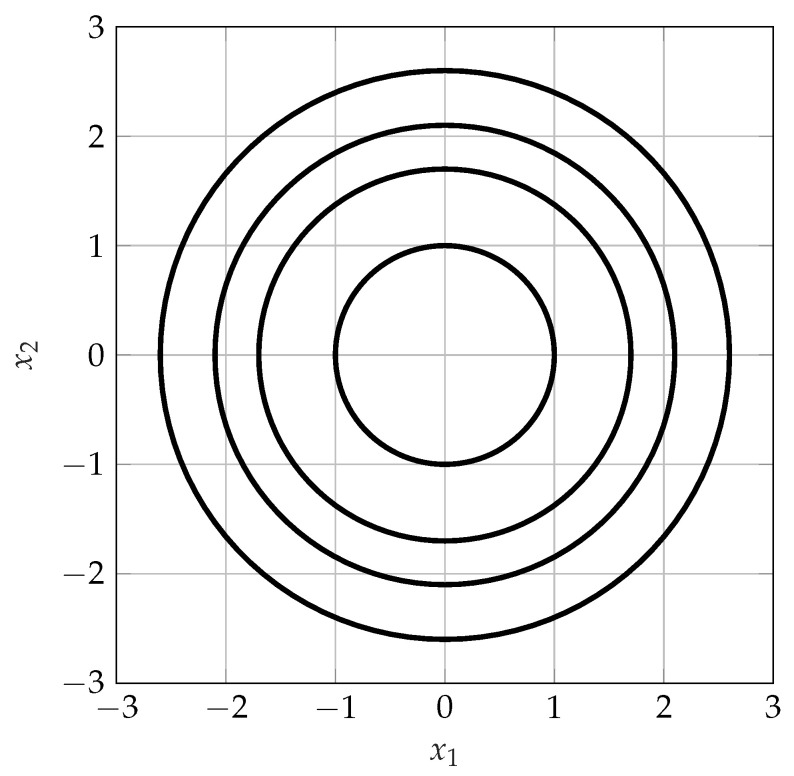
An example of a support of an optimal input distribution for the special case $n_{t}=n_{r}=n=2$.

*As an example consider the special case $n_{r}=n_{t}=n$ with $H=I_{n}$. Then, the union in Equation (4) implies that the cardinality of $E(F_{X}^{⋆})$ is uncountable and that discrete inputs are in general not optimal. Therefore, Theorem 3 can generally not be improved in the sense that for n>1, statements about the cardinality of $E(F_{X}^{⋆})$ cannot be made. Note, however, that the magnitude of X is discrete. An example of the corresponding optimal input distribution for the case of n=2 is given in Figure 1.*

Even though Theorem 3 does not allow us to conclude that the optimal input distribution of an arbitrary MIMO channel is discrete and finite, for the special case of a SISO channel we have the following partial result.

**Theorem** **4.** (Optimal Input Distribution of a SISO Channel [3,6,28]) *For some fixed h∈R and $A\in R_{+}$, consider the SISO channel Y=hX+Z with input space X=[−A,A]. Let $F_{X}^{⋆}$ be an input distribution that achieves the capacity, C(X,h), of that channel. Then, $F_{X}^{⋆}$ satisfies the following properties:*

- $F_{X}^{⋆}$ is unique.
- $F_{X}^{⋆}$ is symmetric.
- $F_{X}^{⋆}$ is discrete with the number of mass points being of the order $O(A^{2})$.
- $F_{X}^{⋆}$ contains probability mass points at {−A,A}.

*Moreover, binary communication with mass points at {−A,A} is optimal if and only if $A\leq \bar{A}$, where $\bar{A}\approx 1.665$.*

Theorem 4 can now be used to also address the special cases of multiple-input single output (MISO) and single-input multiple output (SIMO) channels.

**Theorem** **5.** Let $Y=h^{T}X^{⋆}+Z$ be a MISO channel with channel matrix $h^{T}\in R^{n_{t}}$ and some optimal input $X^{⋆}\in X\subset R^{n_{t}}$. Then, the distribution of $h^{T}X^{*}$ is discrete with finitely many mass points. On the other hand, let $Y=hX^{⋆}+Z$ be a SIMO channel with channel matrix $h\in R^{n_{r}}$. Then, the optimal input $X^{⋆}\in X\subset R$ has a discrete distribution with finitely many mass points.

**Proof.**  For the MISO case, the capacity can expressed as

(5) $$\begin{array}{cc} C(X,h^{T}) & =\max_{F_{X}:X\in X}I\left(X;h^{T}X+Z\right)\\ & =\max_{F_{X}:X\in X}I\left(h^{T}X;h^{T}X+Z\right)\\ & =\max_{F_{S}:S\in h^{T}X}I\left(S;S+Z\right)\\ & =\max_{F_{S}:\left|S\right|\leq r_{\max}\left(h^{T}X\right)}I\left(S;S+Z\right). \end{array}$$

Using Theorem 5 we have that the maximizing distribution in Equation (5) $F_{S}^{⋆}$, where $S:=h^{T}X$, is discrete with finitely many mass points.

For the SIMO case, the channel input distribution is discrete as a SIMO channel can be transformed into a SISO channel. Thus, let $A\in R_{+}$ be finite. Then, the capacity of the SIMO channel can be expressed as

(6) $$\begin{array}{cc} C(X,h) & =\max_{F_{X}:\left|X\right|\leq A}I\left(X;hX+Z\right)\\ & =\max_{F_{X}:\left|X\right|\leq A}I\left(X;∥h∥X+Z\right). \end{array}$$

Again, it follows from Theorem 5 that the mutual information in Equation (6) is maximized by a channel input distribution, $F_{X}^{⋆}$, that is discrete with finitely many mass points. This concludes the proof. □

**Remark** **5.** *Note that in the MISO case, we do not claim $F_{X}^{⋆}$ to be discrete with finitely many points. To illustrate the difficulty, let $h^{T}=\left[1,-1\right]$ so that*

$$h^{T}X^{⋆}=h^{T}\left(\begin{array}{c} X_{1}^{⋆}\\ X_{2}^{⋆} \end{array}\right)=X_{1}^{⋆}-X_{2}^{⋆}.$$

*As $X_{1}^{⋆}$ and $X_{2}^{⋆}$ can be arbitrarily correlated, we cannot rule out cases in which $X_{1}^{⋆}=X-D$ and $X_{2}^{⋆}=X-2D$, with D a discrete random variable and X of arbitrary distribution. Clearly the distribution of $X^{⋆}$ is not discrete.*

Note that in general it can be shown that the capacity-achieving input distribution is discrete if the optimization problem in Equation (2) can be reformulated as an optimization over one dimensional distributions. This, for example, has been done in [5] for parallel channels with a total amplitude constraint.

### 3.3. Properties of Capacity-Achieving Input Distributions in the Small (But Not Vanishing) Amplitude Regime

In this subsection, we study properties of capacity-achieving input distribution in the regime of small amplitudes. To that end, we will need the notion of a subharmonic function.

**Definition** **3.** (Subharmonic Function) *Let f be a real-valued function that is twice continuously differentiable on an open set $G\subset R^{n}$. Then, f is* subharmonic *if $\nabla^{2}f\geq 0$ on G, where $\nabla^{2}$ denotes the Laplacian (if f is twice differentiable, its Laplacian is given by $\nabla^{2}f\left(x_{1},\cdots ,x_{n}\right)=\sum_{i=1}^{n}\frac{\partial^{2}f}{\partial x_{i}^{2}}$).*

We use the following Theorem, which states that a subharmonic function always attains its maximum on the boundary of its domain.

**Theorem** **6.** (Maximum Principle of Subharmonic Functions [29]) *Let $G\subset R^{n}$ be a connected open set. If f:G→R is subharmonic and attains a global maximum in the interior of G, then f is constant on G.*

In addition to Theorem 6, we need the following result that has been proven in [30].

**Lemma** **1.** *Let the likelihood function of the output of a MIMO channel be defined as*

$$\ell :R^{n_{r}}\to R\;,\;\ell \left(y\right)=\frac{f_{Y}\left(y\right)}{\frac{1}{{\left(2\pi \right)}^{\frac{n_{r}}{2}}}e^{-\frac{{∥y∥}^{2}}{2}}}$$

*and let $A_{y}$ denote the Hessian matrix of $\log_{e}\left(\ell (y)\right)$. Then, the Laplacian (or the trace of $A_{y}$) is given by*

(7) $$\nabla^{2}\log_{e}\left(\ell (y)\right)=\mathrm{Tr}\left(A_{y}\right)=E\left[{∥HX∥}^{2}|Y=y\right]-{∥E[HX|Y=y]∥}^{2}.$$

**Theorem** **7.** *Suppose that $r_{\max}^{2}\left(HX\right)\leq \log_{2}\left(e\right)$. Then, $x\mapsto h(x;F_{X})$ is a subharmonic function for every $F_{X}$.*

**Proof.**  Let $F_{X}$ be arbitrary and observe that

$$\begin{array}{cc} h(x;F_{X}) & =-E\left[\log_{2}\left(f_{Y}\left(Hx+Z\right)\right)\right]\\ & =-E\left[\ell (Hx+Z)\right]-E\;\left[\log_{2}\left(\frac{1}{{\left(2\pi \right)}^{\frac{n_{r}}{2}}}e^{-\frac{{∥Hx+Z∥}^{2}}{2}}\right)\right]\\ & =-E\left[\ell (Hx+Z)\right]+E\;\left[\frac{{∥Hx+Z∥}^{2}}{2}\right]\log_{2}\left(e\right)+\log_{2}\left({\left(2\pi \right)}^{\frac{n_{r}}{2}}\right)\\ & =-E\left[\ell (Hx+Z)\right]+\frac{{∥Hx∥}^{2}+n_{r}}{2}\log_{2}\left(e\right)+\log_{2}\left({\left(2\pi \right)}^{\frac{n_{r}}{2}}\right). \end{array}$$

With this expression in hand, the Laplacian of $h(x;F_{X})$ with respect to x can be bounded from below as follows:

(8) $$\begin{array}{cc} \nabla^{2}h\left(x;F_{X}\right) & =\nabla^{2}\left(-E\left[\ell (Hx+Z)\right]+\frac{{∥Hx∥}^{2}+n_{r}}{2}\log_{2}\left(e\right)+\log_{2}\left({\left(2\pi \right)}^{\frac{n_{r}}{2}}\right)\right)\\ & =-E\left[\nabla^{2}\ell \left(Hx+Z\right)\right]+\nabla^{2}\frac{{∥Hx∥}^{2}}{2}\log_{2}\left(e\right)\\ & \overset{\left(a\right)}{=}-E\left[\mathrm{Tr}\left(HH^{T}A_{Hx+Z}\right)\right]+\nabla^{2}\frac{{∥Hx∥}^{2}}{2}\log_{2}\left(e\right)\\ & =-E\left[\mathrm{Tr}\left(HH^{T}A_{Hx+Z}\right)\right]+\mathrm{Tr}\left(HH^{T}\right)\log_{2}\left(e\right)\\ & \overset{\left(b\right)}{\geq }-E\left[\mathrm{Tr}\left(HH^{T}\right)\mathrm{Tr}\left(A_{Hx+Z}\right)\right]+\mathrm{Tr}\left(HH^{T}\right)\log_{2}\left(e\right)\\ & =\mathrm{Tr}\left(HH^{T}\right)\left(-E\left[\mathrm{Tr}\left(A_{Hx+Z}\right)\right]+\log\left(e\right)\right)\\ & \overset{\left(c\right)}{\geq }\mathrm{Tr}\left(HH^{T}\right)\left(-r_{\max}^{2}\left(HX\right)+\log\left(e\right)\right), \end{array}$$

where (a) follows from Equation (7) and the chain rule for the Hessian; (b) from using the well-known inequality

$$\mathrm{Tr}{\left(CD\right)}^{2}\leq \mathrm{Tr}{\left(C\right)}^{2}\mathrm{Tr}{\left(D\right)}^{2}$$

that holds for C and D positive semi-definite; and (c) from using the inequality

$$\begin{array}{cc} \mathrm{Tr}\left(A_{y}\right) & =E\left[{∥HX∥}^{2}|Y=y\right]-{∥E[HX|Y=y]∥}^{2}\\ & \leq E\left[{∥HX∥}^{2}|Y=y\right]\\ & \leq r_{\max}^{2}\left(HX\right). \end{array}$$

Thus, according to the assumption that $r_{\max}^{2}\left(HX\right)\leq \log_{2}\left(e\right)$, the right-hand side of Equation (8) is nonnegative, which proves the result. □

Now, knowing that $h(x;F_{X})$ is a subharmonic function allows us to characterize the support of an optimal input distribution of a MIMO channel provided that the radius of the channel input space, X, is sufficiently small.

**Theorem** **8.** *Let $F_{X}^{⋆}$ be a capacity-achieving input distribution and $r_{\max}^{2}\left(HX\right)\leq \log_{2}\left(e\right)$. Then, $E(F_{X}^{⋆})\subseteq \partial X$.*

**Proof.**  From the KKT conditions in Equation (3), we know that, if $x\in E(F_{X}^{⋆})$, then x is a maximizer of $h(x;F_{X}^{⋆})$. According to Theorem 7, we also know that $h(x;F_{X}^{⋆})$ is subharmonic. Hence, from the Maximum Principle of Subharmonic Functions (i.e., Theorem 6), it follows $E(F_{X}^{⋆})\subseteq \partial X$. □

Combining Theorem 8 with the observations made in Remark 4 leads to the following corollary.

**Corollary** **1.** *Let $X=B_{0}\left(A\right)$ and $A\leq \frac{1}{∥H∥}\log_{2}\left(e\right)$. Then,*

$$E\left(F_{X}^{⋆}\right)\subseteq C\left(A\right),$$

*where $C\left(A\right):=\{x\in R^{n}:∥x∥=A\}$ denotes a sphere of radius A.*

We conclude this section by noting that for the special case $n_{t}=n_{r}=n$ with $H=I_{n}$, the exact value of *A* such that $E\left(F_{X}^{⋆}\right)=C\left(A\right)$ has been characterized in terms of an integral equation in [31], which is approximately equal to $1.5\sqrt{n}$.

## 4. Upper and Lower Bounds on the Capacity

The considerations in the previous section have shown that characterizing the structure of an optimal channel-input distribution is a challenging question in itself that we could only partially answer. A full characterization, however, is a necessary prerequisite to narrow down the search space in Equation (2) to one that is tractable. Except for some special cases (i.e., special choices of X), optimizing over the most general space of input distributions that consists of *all* continuous $n_{t}$-dimensional probability distributions $F_{X}$ with X∈X, is prohibitive (Note that Dytso et al. [32] summarized methods of how to optimize functionals over the space of probability distributions that are constrained in there support). Thus, up to the writing of this paper, there is little hope in being able to solve the problem in Equation (2) in full generality so that in this section we are proposing novel lower and upper bounds on the capacity C(X,H). Nevertheless, these bounds will allow us to better understand how the capacity of such MIMO channels behaves.

Towards this end, in Section 4.1, we provide four upper bounds. The first is based on an upper bound on the differential entropy of a random vector that is constraint in its *p*th moment, the second and third bounds are based on duality arguments, and the fourth on the relationship between mutual information and the minimum mean square error (MMSE), I-MMSE relationship for short, known from [33]. The three lower bounds proposed in Section 4.2, on the other hand, are based on the celebrated entropy power inequality, a generalization of the Ozarow–Wyner capacity bound taken from [11], and on Jensen’s inequality.

### 4.1. Upper Bounds

To establish our first upper bound on Equation (2), we need the following result ([11] Th. 1).

**Lemma** **2.** (Maximum Entropy Under *p*th Moment Constraint) *Let n∈N and p∈(0,∞) be arbitrary. Then, for any $U\in R^{n}$ such that h(U)<∞ and ${∥U∥}_{p}<\infty$, we have*

$$h\left(U\right)\leq n\log_{2}\left(k_{n,p}\;n^{\frac{1}{p}}\;{∥U∥}_{p}\right),$$

*where*

$$k_{n,p}:=\frac{\sqrt{\pi }\;e^{\frac{1}{p}}{\left(\frac{p}{n}\right)}^{\frac{1}{p}}\Gamma {\left(\frac{n}{p}+1\right)}^{\frac{1}{n}}}{\Gamma {\left(\frac{n}{2}+1\right)}^{\frac{1}{n}}}.$$

**Theorem** **9.** (Moment Upper Bound) *For any channel input space X and any fixed channel matrix H, we have*

$$\begin{array}{c} C\left(X,H\right)\leq {\bar{C}}_{M}\left(X,H\right):=\underset{p>0}{\inf}n_{r}\log_{2}\left(\;\frac{k_{n_{r},p}}{{\left(2\pi e\right)}^{\frac{1}{2}}}\;n_{r}^{\frac{1}{p}}{∥\tilde{x}+Z∥}_{p}\right), \end{array}$$

*where $\tilde{x}\in HX$ is chosen such that $∥\tilde{x}∥=r_{\max}\left(HX\right)$.*

**Proof.**  Expressing Equation (2) in terms of differential entropies results in

(9) $$\begin{array}{cc} \;C(X,H) & =\max_{F_{X}:X\in X}h\left(HX+Z\right)-h\left(Z\right)\\ & \overset{\left(a\right)}{\leq }\max_{F_{X}:X\in X}n_{r}\log_{2}\left(\frac{k_{n_{r},p}}{{\left(2\pi e\right)}^{\frac{1}{2}}}\;n_{r}^{\frac{1}{p}}\;{∥HX+Z∥}_{p}\right)\\ & \overset{\left(b\right)}{=}n_{r}\log_{2}\left(\frac{k_{n_{r},p}}{{\left(2\pi e\right)}^{\frac{1}{2}}}\;n_{r}^{\frac{1}{p}}\max_{F_{X}:X\in X}{∥HX+Z∥}_{p}\right)\;, \end{array}$$

where (a) follows from Lemma 2 with the fact that $h\left(Z\right)=\frac{n_{r}}{2}\log_{2}\left(2\pi e\right)$; and (b) from the monotonicity of the logarithm.

Now, notice that ${∥HX+Z∥}_{p}$ is linear in $F_{X}$ and therefore it attains its maximum at an extreme point of the set $F_{X}:=\left\{F_{X}:X\in X\right\}$ (i.e., the set of all cumulative distribution functions of X). As a matter of fact [26], the extreme points of $F_{X}$ are given by the set of degenerate distributions on X; that is, ${\left\{F_{X}\left(y\right)=\delta_{x}\left(y\right),y\in X\right\}}_{x\in X}$. This allows us to conclude

$$\max_{F_{X}:X\in X}{∥HX+Z∥}_{p}=\max_{x\in X}{∥Hx+Z∥}_{p}.$$

Observe that the Euclidian norm is a convex function, which is therefore maximized at the boundary of the set HX. Combining this with Equation (9) and taking the infimum over p>0 completes the proof. □

The following Theorem provides two alternative upper bounds that are based on duality arguments.

**Theorem** **10.** (Duality Upper Bounds) *For any channel input space X and any fixed channel matrix H*

(10) $$C\left(X,H\right)\leq {\bar{C}}_{\mathrm{Dual},1}\left(X,H\right):=\log_{2}\left(c_{n_{r}}\left(d\right)+\frac{\mathrm{Vol}\left(B_{0}\left(d\right)\right)}{{\left(2\pi e\right)}^{\frac{n_{r}}{2}}}\right),$$

*where*

d:=rmax(HX),cnr(d):=∑i=1nr−1nr−1iΓnr−122nr2Γnr2di,

*and*

(11) $$C\left(X,H\right)\leq {\bar{C}}_{\mathrm{Dual},2}\left(X,H\right):=\sum_{i=1}^{n_{r}}\log_{2}\left(1+\frac{2A_{i}}{\sqrt{2\pi e}}\right),$$

*where $a=(A_{1},\cdots ,A_{n_{r}})$ such that Box(a)=Box(HX).*

**Proof.**  Using duality bounds, it has been shown in [8] that for any centered *n*-dimensional ball of radius $r\in R_{+}$

(12) $$\max_{F_{X}:X\in B_{0}\left(r\right)}I\left(X;X+Z\right)\leq \log_{2}\left(c_{n}\left(r\right)+\frac{\mathrm{Vol}\left(B_{0}\left(r\right)\right)}{{\left(2\pi e\right)}^{\frac{n}{2}}}\right),$$

where cn(r):=∑i=1n−1n−1iΓn−122n2Γn2ri.

Now, observe that

(13) $$\begin{array}{cc} C(X,H) & =\max_{F_{X}:X\in X}h\left(HX+Z\right)-h\left(HX+Z|HX\right)\\ & =\max_{F_{X}:X\in X}I\left(HX;HX+Z\right)\\ & =\max_{F_{\tilde{X}}:\tilde{X}\in HX}I\left(\tilde{X};\tilde{X}+Z\right)\\ & \overset{\left(a\right)}{\leq }\max_{F_{\tilde{X}}:\tilde{X}\in B_{0}\left(d\right),d:=r_{\max}\left(HX\right)}I\left(\tilde{X};\tilde{X}+Z\right)\\ & \overset{\left(b\right)}{\leq }\log_{2}\left(c_{n_{r}}\left(d\right)+\frac{\mathrm{Vol}\left(B_{0}\left(d\right)\right)}{{\left(2\pi e\right)}^{\frac{n_{r}}{2}}}\right). \end{array}$$

where (a) follows from enlarging the optimization domain; and (b) from using the upper bound in Equation (12). This proves Equation (10).

To show the upper bound in Equation (11), we proceed with an alternative upper bound to Equation (13):

$$\begin{array}{cc} C(X,H) & =\max_{F_{\tilde{X}}:\tilde{X}\in HX}I\left(\tilde{X};\tilde{X}+Z\right)\\ & \overset{\left(a\right)}{\leq }\max_{F_{\tilde{X}}:\tilde{X}\in \mathrm{Box}\left(HX\right)}I\left(\tilde{X};\tilde{X}+Z\right)\\ & \overset{\left(b\right)}{\leq }\max_{F_{\tilde{X}}:\tilde{X}\in \mathrm{Box}\left(HX\right)}\sum_{i=1}^{n_{r}}I\left({\tilde{X}}_{i};{\tilde{X}}_{i}+Z_{i}\right)\\ & \overset{\left(c\right)}{=}\sum_{i=1}^{n_{r}}\max_{F_{{\tilde{X}}_{i}}:\left|{\tilde{X}}_{i}\right|\leq A_{i}}I\left({\tilde{X}}_{i};{\tilde{X}}_{i}+Z_{i}\right)\\ & \overset{\left(d\right)}{\leq }\sum_{i=1}^{n_{r}}\log_{2}\left(1+\frac{2A_{i}}{\sqrt{2\pi e}}\right), \end{array}$$

where the (in)equalities follow from: (a) enlarging the optimization domain; (b) single-letterizing the mutual information; (c) choosing individual amplitude constraints $\left(A_{1},\cdots ,A_{n_{r}}\right)a\in R_{+}^{n_{r}}$ such that Box(a)=Box(HX); and (d) using the upper bound in Equation (12) for n=1. This concludes the proof. □

As mentioned at the beginning of the section, another simple technique for deriving upper bounds on the capacity is to use the I-MMSE relationship [33]

(14) $$I\left(X;X+Z\right)=\frac{\log_{2}\left(e\right)}{2}\int_{0}^{1}E\left[{∥X-E[X\;|\sqrt{\gamma }X+Z]∥}^{2}\right]d\gamma .$$

For any γ≥0, the quantity $E\left[∥X-E\left[X\;|\sqrt{\gamma }X+Z\right]{∥}^{2}\right]$ is known as the MMSE of estimating X from the noisy observation $\sqrt{\gamma }X+Z$. An important fact that will be useful is that the conditional expected value $E[X\;|\sqrt{\gamma }X+Z]$ is the best estimator in the sense that it minimizes the mean square error over all measurable functions $f:R^{n_{r}}\to R^{n_{t}}$; that is, for any $Y\in R^{n_{r}}$ and $X\in R^{n_{t}}$

(15) $$E\left[{∥X-E\left[X|Y\right]∥}^{2}\right]=\underset{f\;\mathrm{is}\;\mathrm{measurable}\;}{\inf}E\left[{∥X-f\left(Y\right)∥}^{2}\right].$$

**Theorem** **11.** (I-MMSE Upper Bound) *For any channel input space X and any fixed channel matrix H*

$$C\left(X,H\right)\leq {\bar{C}}_{I-\mathrm{MMSE}}\left(X,H\right)=\log_{2}\left(e\right)\left\{\begin{array}{cc} \frac{n_{r}}{2}+\frac{n_{r}}{2}\log_{2}\left(\frac{r_{\max}^{2}\left(HX\right)}{n_{r}}\right), & r_{\max}^{2}(HX\geq n_{r}\\ \frac{r_{\max}^{2}\left(HX\right)}{2}, & r_{\max}^{2}\left(HX\right)\leq n_{r} \end{array}\right..$$

**Proof.**  Fix some ϵ∈[0,1] and observe that

$$\begin{array}{cc} \frac{2}{\log_{2}\left(e\right)}I\left(X;HX+Z\right) & \overset{\left(a\right)}{=}\frac{2}{\log_{2}\left(e\right)}I\left(HX;HX+Z\right)\\ & \overset{\left(b\right)}{=}\int_{0}^{1}E\left[∥HX-E\left[HX|\sqrt{\gamma }HX+Z\right]{∥}^{2}\right]d\gamma \\ & =\int_{0}^{\epsilon }E\left[∥HX-E\left[HX|\sqrt{\gamma }HX+Z\right]{∥}^{2}\right]d\gamma \\ & +\int_{\epsilon }^{1}E\left[∥HX-E\left[HX|\sqrt{\gamma }HX+Z\right]{∥}^{2}\right]d\gamma \\ & \overset{\left(c\right)}{\leq }\int_{0}^{\epsilon }E\left[{∥HX-0∥}^{2}\right]d\gamma \\ & +\int_{\epsilon }^{1}E\left[{∥HX-\frac{1}{\sqrt{\gamma }}\left(\sqrt{\gamma }HX+Z\right)∥}^{2}\right]d\gamma \\ & =\epsilon \;E\left[{∥HX∥}^{2}\right]+\int_{\epsilon }^{1}\frac{1}{\gamma }E\left[{∥Z∥}^{2}\right]d\gamma \\ & =\epsilon \;E\left[{∥HX∥}^{2}\right]+E\left[{∥Z∥}^{2}\right]\log_{e}\left(\frac{1}{\epsilon }\right)\\ & =\epsilon \;E\left[{∥HX∥}^{2}\right]+n_{r}\log_{e}\left(\frac{1}{\epsilon }\right), \end{array}$$

where the (in)equalities follow from: (a) using that I(HX;HX+Z)=I(X;HX+Z) for any fixed H; (b) using the I-MMSE relationship in Equation (14); and (c) using the property that conditional expectation minimizes mean square error (i.e., (15)).

Now, notice that

(16) $$\begin{array}{cc} \max_{F_{X}:X\in X}I\left(X;HX+Z\right) & \leq \max_{F_{X}:X\in X}\frac{\log_{2}\left(e\right)}{2}\left(\epsilon \;E\left[{∥HX∥}^{2}\right]+n_{r}\log_{e}\left(\frac{1}{\epsilon }\right)\right)\\ & \overset{\left(a\right)}{=}\frac{1}{2}\left(\epsilon \;\max_{\tilde{x}\in X}{∥H\tilde{x}∥}^{2}+n_{r}\log_{e}\left(\frac{1}{\epsilon }\right)\right)\\ & \overset{\left(b\right)}{=}\frac{1}{2}\left(\epsilon \;r_{\max}^{2}\left(HX\right)+n_{r}\log_{e}\left(\frac{1}{\epsilon }\right)\right), \end{array}$$

where (a) follows from $\max_{F_{X}:X\in X}E\left[{∥HX∥}^{2}\right]=\max_{\tilde{x}\in X}{∥H\tilde{x}∥}^{2}$ (the same argument was used in the proof of Theorem 9); and (b) from the definition of $r_{\max}^{2}\left(HX\right)$.

Since ϵ is arbitrary, we can choose it to minimize the upper bound in Equation (16). Towards this end, we need the following optimization result

(17) $$\min_{\epsilon \in [0,1]}\left(\epsilon \;a+b\log\left(\frac{1}{\epsilon }\right)\right)=\left\{\begin{array}{cc} b+b\log\left(\frac{a}{b}\right), & a\geq b\\ a, & a\leq b \end{array}\right.,$$

which is easy to show. Combining Equation (16) with Equation (17), we obtain the following upper bound on the capacity

$$\max_{F_{X}:X\in X}I\left(X;HX+Z\right)\leq \left\{\begin{array}{cc} \frac{n_{r}}{2}+\frac{n_{r}}{2}\log\left(\frac{r_{\max}^{2}\left(HX\right)}{n_{r}}\right), & r_{\max}^{2}\left(HX\right)\geq n_{r}\\ r_{\max}^{2}\left(HX\right), & r_{\max}^{2}\left(HX\right)\leq n_{r} \end{array}\right..$$

This concludes the proof. □

**Corollary** **2.** *For any channel input space X and any fixed channel matrix H*

$${\bar{C}}_{I-\mathrm{MMSE}}\left(X,H\right)\leq \log_{2}\left(e\right)\left\{\begin{array}{cc} \frac{n_{r}}{2}+\frac{n_{r}}{2}\log_{e}\left(\frac{{∥H∥}^{2}r_{\max}^{2}\left(X\right)}{n_{r}}\right), & {∥H∥}^{2}r_{\max}^{2}\left(X\right)\geq n_{r}\\ \frac{{∥H∥}^{2}r_{\max}^{2}\left(X\right)}{2}, & {∥H∥}^{2}r_{\max}^{2}\left(X\right)\leq n_{r} \end{array}\right..$$

**Proof.**  The corollary follows by upper bounding Equation (16) using the fact that $r_{\max}^{2}\left(HX\right)\leq ∥H∥r_{\max}^{2}\left(X\right)$. □

**Remark** **6.** *In the proof of Theorem 11, instead of using sub-optimal estimators f(Y)=0 and $f\left(Y\right)=\frac{1}{\gamma }Y$, we could have used an optimal linear estimator of the form $f\left(Y\right)=K_{XY}K_{Y}^{-1}Y$, where $K_{XY}$ denotes the cross-covariance matrix between X and Y and $K_{Y}$ the covariance matrix of Y. This choice would result in the capacity upper bound*

(18) $$C\left(X,H\right)\leq \max_{K_{X}:X\in X}\frac{1}{2}\log_{2}\left(\det\left(I_{n_{r}}+HK_{X}H^{T}\right)\right)$$

*with $K_{X}$ the covariance matrix of the channel input. While Equation (18) is a valid upper bound, as of the writing of this paper, it is not clear how to perform an optimization over covariance matrices of random variables with bounded support. One possibility to avoid this is to use the inequality between arithmetic and geometric mean and bound the determinant by the trace:*

(19) $$\det\left(I_{n_{r}}+HK_{X}H^{T}\right)\leq {\left(\frac{\mathrm{Tr}\left(I_{n_{r}}+HK_{X}H^{T}\right)}{n_{r}}\right)}^{n_{r}}={\left(\frac{{∥Z+HX∥}_{2}^{2}}{n_{r}}\right)}^{n_{r}}.$$

*However, combining Equation (19) with Equation (18) is merely a special case of the moment upper bound of Theorem 9 for p=2. Therefore, the estimators in Theorem 11 are chosen to obtain a non-trivial upper bound avoiding the optimization over covariance matrices.*

In Section 5, we present a comparison of the upper bounds of Theorems 9–11 by means of a simple example.

### 4.2. Lower Bounds

A classical approach to bound a mutual information from below is to use the entropy power inequality (EPI).

**Theorem** **12.** (EPI Lower Bounds) *For any fixed channel matrix H and any channel input space X with X absolutely continuous, we have*

(20) $$C\left(X,H\right)\geq {\underline{C}}_{\mathrm{EPI}}\left(X,H\right):=\max_{F_{X}:X\in X}\frac{n_{r}}{2}\log_{2}\left(\;1+\frac{2^{\frac{2}{n_{r}}h\left(HX\right)}}{2\pi e}\;\right).$$

*Moreover, if $n_{t}=n_{r}=n$, $H\in R^{n\times n}$ invertible, and X uniformly distributed over X, then*

(21) $$C\left(X,H\right)\geq {\underline{C}}_{\mathrm{EPI}}\left(X,H\right):=\frac{n}{2}\log_{2}\left(\;1+\frac{{\left|\det\left(H\right)\right|}^{\frac{2}{n}}\mathrm{Vol}{\left(X\right)}^{\frac{2}{n}}}{2\pi e}\;\right).$$

**Proof.**  By means of the EPI

$$2^{\frac{2}{n_{r}}h\left(HX+Z\right)}\geq 2^{\frac{2}{n_{r}}h\left(HX\right)}+2^{\frac{2}{n_{r}}h\left(Z\right)},$$

we conclude

$$2^{\frac{2}{n_{r}}C\left(X,H\right)}\geq 1+{\left(2\pi e\right)}^{-1}2^{\frac{2}{n_{r}}\max_{F_{X}:X\in X}h\left(HX\right)},$$

which finalizes the proof of the lower bound in Equation (20).

To show the lower bound in Equation (21), all we need is to recall that

$$h\left(HX\right)=h\left(X\right)+\log_{2}\left|\det\left(H\right)\right|,$$

which is maximized for X uniformly distributed over X. However, if X is uniformly drawn from X, we have

$$2^{\frac{2}{n}h\left(HX\right)}=\mathrm{Vol}{\left(HX\right)}^{\frac{2}{n}}={\left|\det\left(H\right)\right|}^{\frac{2}{n}}\mathrm{Vol}{\left(X\right)}^{\frac{2}{n}},$$

which completes the proof. □

The considerations in Section 3 suggest that a channel input distribution that maximizes Equation (2) might be discrete. Therefore, there is a need for lower bounds that unlike the bounds in Theorem 12 rely on discrete inputs.

**Theorem** **13.** (Ozarow–Wyner Type Lower Bound) *Let $X_{D}\in \mathrm{supp}\left(X_{D}\right)\subset R^{n_{t}}$ be a discrete random vector of finite entropy, $g:R^{n_{r}}\to R^{n_{t}}$ a measurable function, and p>0. Furthermore, let $K_{p}$ be a set of continuous random vectors, independent of $X_{D}$, such that for every $U\in K_{p}$ we have h(U)<∞, ${∥U∥}_{p}<\infty$, and*

(22) $$\mathrm{supp}\left(U+x_{i}\right)\cap \mathrm{supp}\left(U+x_{j}\right)=⌀$$

*for all $x_{i},x_{j}\in \mathrm{supp}\left(X_{D}\right)$, i≠j. Then,*

$$C\left(X,H\right)\geq {\underline{C}}_{\mathrm{OW}}\left(X,H\right):={\left[H\left(X_{D}\right)-\mathrm{gap}\right]}^{+},$$

*where*

$$\mathrm{gap}:=\underset{\begin{array}{c} U\in K_{p}\\ g\;measurable\\ p>0 \end{array}}{\inf}\left(G_{1,p}\left(U,X_{D},g\right)+G_{2,p}\left(U\right)\right)$$

*with*

(23) $$\begin{array}{cc} G_{1,p}\left(U,X_{D},g\right) & :=n_{t}\log_{2}\left(\frac{∥U+X_{D}{-g\left(Y\right)∥}_{p}}{{∥U∥}_{p}}\right), \end{array}$$

(24) $$\begin{array}{cc} G_{2,p}\left(U\right) & :=n_{t}\log_{2}\left(\frac{k_{n_{t},p}\;n_{t}^{\frac{1}{p}}\;{∥U∥}_{p}}{2^{\frac{1}{n_{t}}h\left(U\right)}}\right), \end{array}$$

*and $k_{n_{t},p}$ as defined in Lemma 2, respectively.*

**Proof.**  The proof is identical to ([11] Theorem 2). To make the manuscript more self-contained, we repeat it here.

Let U and $X_{D}$ be statistically independent. Then, the mutual information $I(X_{D};Y)$ can be lower bounded as

(25) $$\begin{array}{cc} I(X_{D};Y) & \overset{\left(a\right)}{\geq }I\left(X_{D}+U;Y\right)\\ & =h\left(X_{D}+U\right)-h\left(X_{D}+U|Y\right)\\ & \overset{\left(b\right)}{=}H\left(X_{D}\right)+h\left(U\right)-h\left(X_{D}+U|Y\right). \end{array}$$

Here, (a) follows from the data processing inequality as $X_{D}+U\to X_{D}\to Y$ forms a Markov chain in that order; and (b) from the assumption in Equation (22). By using Lemma 2, we have that the last term in Equation (25) can be bounded from above as

$$h\left(X_{D}+U|Y\right)\leq n_{t}\log_{2}\left(k_{n_{t},p}\;n_{t}^{\frac{1}{p}}\;{∥X_{D}+U-g\left(Y\right)∥}_{p}\right).$$

Combining this expression with Equation (25) results in

$$I\left(X_{D};Y\right)\geq H\left(X_{D}\right)-\left(G_{1,p}\left(U,X_{D},g\right)+G_{2,p}\left(U\right)\right),$$

with $G_{1,p}$ and $G_{2,p}$ as defined in Equations (23) and (24), respectively. Maximizing the right-hand side over all $U\in K_{p}$, measurable functions $g:R^{n_{r}}\to R^{n_{t}}$, and p>0 provides the bound. □

Interestingly, the bound of Theorem 13 holds for arbitrary channels and is therefore not restricted to MIMO channels. The interested reader is referred to [11] for details.

We conclude the section by providing a lower bound that is based on Jensen’s inequality and holds for arbitrary inputs.

**Theorem** **14.** (Jensen’s Inequality Lower Bound) *For any channel input space X and any fixed channel matrix H, we have*

(26) $$C\left(X,H\right)\geq {\underline{C}}_{\mathrm{Jensen}}\left(X,H\right):=\max_{F_{X}:X\in X}\log_{2}^{+}\left({\left(\frac{2}{e}\right)}^{\frac{n_{r}}{2}}\psi^{-1}\left(X,H\right)\right)$$

*with*

$$\psi \left(X,H\right):=E[\exp\left(-\frac{∥H\left(X-X^{\prime }\right){∥}^{2}}{4}\right)]=E\;\left[{\left|\phi_{X}\left(\frac{H^{T}Z}{\sqrt{2}}\right)\right|}^{2}\right],$$

*where $X^{\prime }$ is an independent copy of X and $\phi_{X}$ denotes the characteristic function of X.*

**Proof.**  To show the lower bound, we follow an approach of Dytso et al. [34]. Note that by Jensen’s inequality

(27) $$h\left(Y\right)=-E\left[\log_{2}f_{Y}\left(Y\right)\right]\geq -\log_{2}E\left[f_{Y}\left(Y\right)\right]=-\log_{2}\int_{R^{n_{r}}}\;f_{Y}\left(y\right)f_{Y}\left(y\right)\;dy.$$

Now, evaluating the integral in Equation (27) results in

(28) $$\begin{array}{cc} \int_{R^{n_{r}}}f_{Y}\left(y\right)f_{Y}\left(y\right)\;dy & =\frac{1}{{\left(2\pi \right)}^{n_{r}}}\int_{R^{n_{r}}}E\left[e^{-\frac{{∥y-HX∥}^{2}}{2}}\right]E\left[e^{-\frac{∥y-HX^{\prime }{∥}^{2}}{2}}\right]dy\\ & \overset{\left(a\right)}{=}\frac{1}{{\left(2\pi \right)}^{n_{r}}}E\left[\int_{R^{n_{r}}}e^{-\frac{{∥y-HX∥}^{2}+{∥y-HX^{\prime }∥}^{2}}{2}}dy\right]\\ & \overset{\left(b\right)}{=}\frac{1}{{\left(2\pi \right)}^{n_{r}}}E\left[e^{-\frac{∥HX-HX^{\prime }{∥}^{2}}{4}}\int_{R^{n_{r}}}e^{-∥y-\frac{H(X-X^{\prime })}{2}{∥}^{2}}dy\right]\\ & \overset{\left(c\right)}{=}\frac{1}{2^{n_{r}}\pi^{\frac{n_{r}}{2}}}E\left[e^{-\frac{∥H\left(X-X^{\prime }\right){∥}^{2}}{4}}\right], \end{array}$$

where (a) follows from the independence of X and $X^{\prime }$ and Tonelli’s Theorem ([35] Chapter 5.9); (b) from completing a square; and (c) from the fact that $\int_{R^{n_{r}}}e^{-∥y-\frac{H(X-X^{\prime })}{2}{∥}^{2}}dy=\int_{R^{n_{r}}}e^{-{∥y∥}^{2}}dy=\pi^{\frac{n_{r}}{2}}$. Combining Equation (27) with Equation (28) and subtracting $h\left(Z\right)=\frac{n_{r}}{2}\log_{2}\left(2\pi e\right)$ completes the proof of the first version of the bound.

To show the second version, observe that

$$\begin{array}{cc} E\left[e^{-\frac{∥H\left(X-X^{\prime }\right){∥}^{2}}{4}}\right] & \overset{\left(d\right)}{=}E\left[\phi_{\frac{H(X-X^{\prime })}{\sqrt{2}}}\left(Z\right)\right]\\ & \overset{\left(e\right)}{=}E\left[\phi_{\frac{HX}{\sqrt{2}}}\left(Z\right)\phi_{-\frac{HX^{\prime }}{\sqrt{2}}}\left(Z\right)\right]\\ & \overset{\left(f\right)}{=}E\;\left[\phi_{X}\left(\frac{H^{T}Z}{\sqrt{2}}\right)\phi_{X^{\prime }}\left(-\frac{H^{T}Z}{\sqrt{2}}\right)\right]\\ & \overset{\left(g\right)}{=}E\;\left[\phi_{X}\left(\frac{H^{T}Z}{\sqrt{2}}\right)\phi_{X}\left(-\frac{H^{T}Z}{\sqrt{2}}\right)\right]\\ & \overset{\left(h\right)}{=}E\;\left[\phi_{X}\left(\frac{H^{T}Z}{\sqrt{2}}\right)\phi_{X}^{*}\left(\frac{H^{T}Z}{\sqrt{2}}\right)\right]\\ & =E\;\left[{\left|\phi_{X}\left(\frac{H^{T}Z}{\sqrt{2}}\right)\right|}^{2}\right], \end{array}$$

where (d) follows from Parseval’s identity ([35] Chapter 9.5) by noting that $\exp(-∥\cdot {∥}^{2}/2)$ is a characteristic function of Z and $\phi_{\frac{H(X-X^{\prime })}{\sqrt{2}}}\left(\cdot \right)$ is a characteristic function of $\frac{H(X-X^{\prime })}{\sqrt{2}}$; (e) from using the property that the characteristic function of a sum of random vectors is equal to the product of its characteristic functions; (f) from using the fact that a characteristic function is a linear transformation; (g) from using that X and $X^{\prime }$ have the same characteristic function; and (h) from the fact that the characteristic function is Hermitian. This completes the proof. □

**Remark** **7.** *As is evident from our examples in the following sections, in many cases, the Jensen’s inequality lower bound of Theorem 14 performs remarkably well. The bound, however, is also useful for MIMO channels that are subject to an average power constraint. For example, evaluating Equation (26) with $X∼N(0,K_{X})$ results in*

$$I\left(X;HX+Z\right)\geq \frac{1}{2}\log_{2}^{+}\left({\left(\frac{2}{e}\right)}^{\min(n_{r},n_{t})}\det\left(I_{n_{r}}+HK_{X}H^{T}\right)\right).$$

*Note that this bound is within $\frac{\min(n_{r},n_{t})}{2}\log_{2}\left(\frac{2}{e}\right)$ bits of the capacity of the power-constrained channel.*

In Section 3, we discuss that the distributions that maximize mutual information in $n_{t}$-dimensions are typically singular, which means that they are concentrated on a set of Lebesgue measure zero. Singular distributions generally do not have a probability density, whereas the characteristic function always exists. This is why the version of Jensen’s inequality lower bound in Theorem 14 that is based on the characteristic function of the channel input is especially useful for amplitude-constrained MIMO channels.

## 5. Invertible Channel Matrices

Consider the symmetric case of $n_{t}=n_{r}=n$ antennas with $H\in R^{n\times n}$ being invertible. In this section, we evaluate some of the lower and upper bounds proposed in the previous section for the special case of H being also diagonal and then characterize the gap to the capacity for arbitrary invertible channel matrices.

### 5.1. Diagonal Channel Matrices

Suppose the channel inputs are subject to per-antenna or an *n*-dimensional amplitude constraint. Then, the duality upper bound ${\bar{C}}_{\mathrm{Dual},2}\left(X,H\right)$ of Theorem 10 takes on the following form.

**Theorem** **15.** (Upper Bounds) *Let $H=\mathrm{diag}\left(h_{11},\cdots ,h_{nn}\right)\in R^{n\times n}$ be fixed. If X=Box(a) for some $a=\left(A_{1},\cdots ,A_{n}\right)\in R_{+}^{n}$, then*

(29) $${\bar{C}}_{\mathrm{Dual},2}\left(\mathrm{Box}\left(a\right),H\right)=\sum_{i=1}^{n}\log_{2}\left(1+\frac{2|h_{ii}|A_{i}}{\sqrt{2\pi e}}\right).$$

*Moreover, if $X=B_{0}\left(A\right)$ for some $A\in R_{+}$, then*

(30) $${\bar{C}}_{\mathrm{Dual},2}\left(B_{0}\left(A\right),H\right)=\sum_{i=1}^{n}\log_{2}\left(1+\frac{2|h_{ii}|A}{\sqrt{n}\sqrt{2\pi e}}\right).$$

**Proof.**  The bound in Equation (29) immediately follows from Theorem 10 by observing that Box(HBox(a))=Box(Ha). The bound in Equation (30) follows from Theorem 10 by the fact that

$$\mathrm{Box}\left(HB_{0}\left(A\right)\right)\subset \mathrm{Box}\left(H\mathrm{Box}\left(B_{0}\left(A\right)\right)\right)=\mathrm{Box}\left(h\right),$$

where $h:=\frac{A}{\sqrt{n}}\left(\right|h_{11}\left|,\cdots ,\right|h_{nn}\left|\right)$. This concludes the proof. □

For an arbitrary channel input space X, the EPI lower bound of Theorem 12 and Jensen’s inequality lower bound of Theorem 14 take on the following form.

**Theorem** **16.** (Lower Bounds) *Let $H=\mathrm{diag}\left(h_{11},\cdots ,h_{nn}\right)\in R^{n\times n}$ be fixed and X arbitrary. Then,*

(31) $${\underline{C}}_{\mathrm{Jensen}}\left(X,H\right)=\log_{2}^{+}\left({\left(\frac{2}{e}\right)}^{\frac{n}{2}}\frac{1}{\psi (H,b^{⋆})}\right)$$

*with*

$$\psi \left(H,b^{⋆}\right):=\min_{b\in X}\prod_{i=1}^{n}\varphi (|h_{ii}\left|B_{i}\right),$$

*where $b:=(B_{1},\cdots ,B_{n})$ and $\varphi :R_{+}\to R_{+}$,*

(32) $$\varphi \left(x\right):=\frac{1}{x^{2}}\left(e^{-x^{2}}-1+\sqrt{\pi }x\left(1-2Q(\sqrt{2}x)\right)\right).$$

*Moreover,*

(33) $${\underline{C}}_{\mathrm{EPI}}\left(X,H\right)=\frac{n}{2}\log_{2}\left(1+\mathrm{Vol}{\left(X\right)}^{\frac{2}{n}}\frac{{\left|\prod_{i=1}^{n}h_{ii}\right|}^{\frac{2}{n}}}{2\pi e}\right).$$

**Proof.**  For some given values $B_{i}\in R_{+}$, i=1,⋯,n, let the *i*th component of $X=(X_{1},\cdots ,X_{n})$ be independent and uniformly distributed over the interval $\left[-B_{i},B_{i}\right]$. Thus, the expected value appearing in the bound of Theorem 14 can be written as

(34) $$E\left[e^{-\frac{∥H\left(X-X^{\prime }\right){∥}^{2}}{4}}\right]=E\left[e^{-\frac{\sum_{i=1}^{n}h_{ii}^{2}{\left(X_{i}-X_{i}^{\prime }\right)}^{2}}{4}}\right]=E\left[\prod_{i=1}^{n}e^{-\frac{h_{ii}^{2}{\left(X_{i}-X_{i}^{\prime }\right)}^{2}}{4}}\right]=\prod_{i=1}^{n}E\left[e^{-\frac{h_{ii}^{2}{\left(X_{i}-X_{i}^{\prime }\right)}^{2}}{4}}\right].$$

Now, if $X^{\prime }$ is an independent copy of X, it can be shown that the expected value at the right-hand side of Equation (34) is of the explicit form

$$E\left[e^{-\frac{h_{ii}^{2}{\left(x_{i}-x_{i}^{\prime }\right)}^{2}}{4}}\right]=\varphi (|h_{ii}\left|B_{i}\right)\;$$

with φ as defined in Equation (32). Finally, optimizing over all $b=(B_{1},\cdots ,B_{n})\in X$ results in the bound (31). The bound in Equation (33) follows by inserting $\left|\det\left(H\right)\right|=\left|\prod_{i=1}^{n}h_{ii}\right|$ into Equation (21), which concludes the proof. □

**Figure 2:**
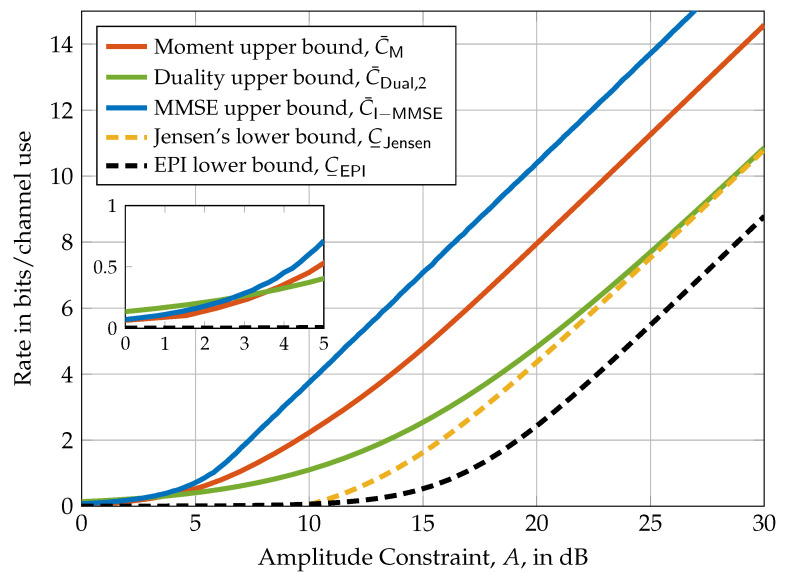
Comparison of the upper and lower bounds of Theorems 9, 11, 15, and 16 evaluated for a 2×2 MIMO system with per-antenna amplitude constraints $A_{1}=A_{2}=A$ (i.e., a=(A,A)) and channel matrix $H=\left(\begin{array}{cc} 0.3 & 0\\ 0 & 0.1 \end{array}\right)$. The nested figure represents a zoom into the region 0dB≤A≤5dB to visualize the differences between the bounds at small amplitude constraints.

In Figure 2, the upper bounds of Theorems 9 and 15 and the lower bounds of Theorem 16 are depicted for a diagonal 2×2 MIMO channel with per-antenna amplitude constraints. It turns out that the moment upper bound and the EPI lower bound perform well in the small amplitude regime while the duality upper bound and Jensen’s inequality lower bound perform well in the high amplitude regime. Interestingly, for this specific example, the duality upper bound and Jensen’s lower bound are asymptotically tight.

### 5.2. Gap to the Capacity

Our first result provides and upper bound to the gap between the capacity in Equation (2) and the lower bound in Equation (21).

**Theorem** **17.** *Let $H\in R^{n\times n}$ be of full rank and*

$$\rho \left(X,H\right):=\frac{\mathrm{Vol}\left(B_{0}\left(r_{\max}\left(HX\right)\right)\right)}{\mathrm{Vol}(HX)}.$$

*Then,*

$$C\left(X,H\right)-{\underline{C}}_{\mathrm{EPI}}\left(X,H\right)\leq \frac{n}{2}\log_{2}\left({\left(\pi n\right)}^{\frac{1}{n}}\rho {\left(X,H\right)}^{\frac{2}{n}}\right).$$

**Proof.**  For notational convenience, let the volume of an *n*-dimensional ball of radius r>0 be denoted as

$$V_{n}\left(r\right):=\mathrm{Vol}\left(B_{0}\left(r\right)\right)=V_{n}\left(1\right)r^{n}=\frac{\pi^{\frac{n}{2}}r^{n}}{\Gamma \left(\frac{n}{2}+1\right)}.$$

Now, observe that, by choosing p=2, the upper bound of Theorem 9 can further be upper bounded as

$$\begin{array}{cc} {\bar{C}}_{M}\left(X,H\right) & \leq n\log_{2}\left(\frac{k_{n,2}}{{\left(2\pi e\right)}^{\frac{1}{2}}}\;n^{\frac{1}{2}}{∥\tilde{x}+Z∥}_{2}\right)\\ & \overset{\left(a\right)}{=}\frac{n}{2}\log_{2}\left(\frac{1}{n}E\left[∥\tilde{x}{+Z∥}^{2}\right]\right)\\ & \overset{\left(b\right)}{=}\frac{n}{2}\log_{2}\left(1+\frac{1}{n}{∥\tilde{x}∥}^{2}\right), \end{array}$$

where (a) follows since $k_{n,2}=\sqrt{\frac{2\pi e}{n}}$; and (b) since $E[∥Z{∥}^{2}]=n$. Therefore, the gap between Equation (21) and the moment upper bound of Theorem 9 can be upper bounded as follows:

$$\begin{array}{cc} {\bar{C}}_{M}\left(X,H\right)-{\underline{C}}_{\mathrm{EPI}}\left(X,H\right) & =\frac{n}{2}\log_{2}\left(\;\frac{1+\frac{1}{n}{∥\tilde{x}∥}^{2}}{1+\frac{\mathrm{Vol}{\left(HX\right)}^{\frac{2}{n}}}{2\pi e}}\;\right)\\ & \overset{a)}{=}\frac{n}{2}\log_{2}\left(\;\frac{1+\frac{1}{n}{\left(\frac{V_{n}(∥\tilde{x}∥)}{V_{n}\left(1\right)}\right)}^{\frac{2}{n}}}{1+\frac{\mathrm{Vol}{\left(HX\right)}^{\frac{2}{n}}}{2\pi e}}\;\right)\\ & =\frac{n}{2}\log_{2}\left(\;\frac{1+\frac{1}{n}{\left(\frac{\rho (X,H)\mathrm{Vol}(HX)}{V_{n}\left(1\right)}\right)}^{\frac{2}{n}}}{1+\frac{\mathrm{Vol}{\left(HX\right)}^{\frac{2}{n}}}{2\pi e}}\;\right)\\ & \overset{b)}{\leq }\frac{n}{2}\log_{2}\left(\;\frac{1}{n}2\pi e{\left(\frac{\rho (X,H)}{V_{n}\left(1\right)}\right)}^{\frac{2}{n}}\;\right)\\ & \overset{c)}{\leq }\frac{n}{2}\log_{2}\left(\;{\left(\pi n\right)}^{\frac{1}{n}}\rho {\left(X,H\right)}^{\frac{2}{n}}\;\right). \end{array}$$

where (a) is due to the fact that $∥\tilde{x}∥$ is the radius of an *n*-dimensional ball; (b) follows from the inequality $\frac{1+cx}{1+x}\leq c$ for c≥1 and $x\in R_{+}$; and (c) follows from using Stirling’s approximation to obtain ${\left(\frac{1}{V_{n}\left(1\right)}\right)}^{\frac{2}{n}}\leq \frac{1}{2e\pi^{1-\frac{1}{n}}}n^{1+\frac{1}{n}}$. □

The term ρ(X,H) is referred to as the *packing efficiency* of the set HX. In the following proposition, we present the packing efficiencies for important special cases.

**Proposition** **1.** (Packing Efficiencies) *Let $H\in R^{n\times n}$ be of full rank, $A\in R_{+}$, and $a:=\left(A_{1},\cdots ,A_{n}\right)\in R_{+}^{n}$. Then,*

(35) $$\begin{array}{cc} \rho \left(B_{0}\left(A\right),I_{n}\right) & =1, \end{array}$$

(36) $$\begin{array}{cc} \rho \left(B_{0}\left(A\right),H\right) & =\frac{{∥H∥}^{n}}{\left|\det(H)\right|}, \end{array}$$

(37) $$\begin{array}{cc} \rho \left(\mathrm{Box}\left(a\right),I_{n}\right) & =\frac{\pi^{\frac{n}{2}}}{\Gamma \left(\frac{n}{2}+1\right)}\frac{{∥a∥}^{n}}{\prod_{i=1}^{n}A_{i}}, \end{array}$$

(38) $$\begin{array}{cc} \rho \left(\mathrm{Box}(a),H\right) & \leq \frac{\pi^{\frac{n}{2}}}{\Gamma \left(\frac{n}{2}+1\right)}\frac{{∥H∥}^{n}{∥a∥}^{n}}{\left|\det\left(H\right)\right|\prod_{i=1}^{n}A_{i}}. \end{array}$$

**Proof.**  The packing efficiency in Equation (35) follows immediately. Note that

$$r_{\max}\left(HB_{0}\left(A\right)\right)=\max_{x\in B_{0}\left(A\right)}∥Hx∥=∥H∥A.$$

Thus, as H is assumed to be invertible, we have $\mathrm{Vol}\left(HB_{0}\left(A\right)\right)=\left|\det\left(H\right)\right|\mathrm{Vol}\left(B_{0}\left(A\right)\right)$, which results in Equation (36). To show Equation (37), observe that

$$\mathrm{Vol}\left(B_{0}\left(r_{\max}\left(I_{n}\mathrm{Box}\left(a\right)\right)\right)\right)=\mathrm{Vol}\left(B_{0}\left(∥a∥\right)\right)=\frac{\pi^{\frac{n}{2}}}{\Gamma \left(\frac{n}{2}+1\right)}{∥a∥}^{n}.$$

The proof of Equation (37) is concluded by observing that $\mathrm{Vol}\left(I_{n}\mathrm{Box}\left(a\right)\right)=\prod_{i=1}^{n}A_{i}$. Finally, observe that $\mathrm{Box}\left(a\right)\subset B_{0}\left(∥a∥\right)$ implies $r_{\max}\left(H\mathrm{Box}\left(a\right)\right)\leq r_{\max}(HB_{0}\left(∥a∥)\right)$ so that

$$\rho \left(H,\mathrm{Box}(a)\right)\leq \frac{\mathrm{Vol}\left(B_{0}\left(∥H∥∥a∥\right)\right)}{\mathrm{Vol}\left(H\mathrm{Box}(a)\right)}=\frac{\pi^{\frac{n}{2}}}{\Gamma \left(\frac{n}{2}+1\right)}\frac{{∥H∥}^{n}{∥a∥}^{n}}{\left|\det\left(H\right)\right|\prod_{i=1}^{n}A_{i}},$$

which is the bound in Equation (38). □

**Figure 3:**
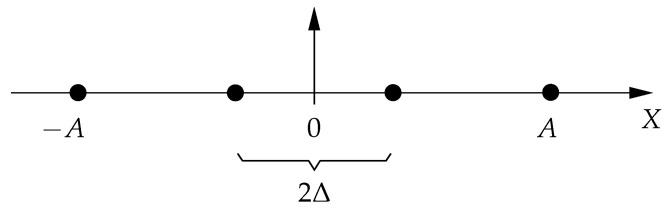
Example of a pulse-amplitude modulation constellation with N=4 points and amplitude constraint *A* (i.e., PAM(4,A)), where Δ:=A/(N−1) denotes half the Euclidean distance between two adjacent constellation points. In the case *N* is odd, 0 is a constellation point.

We conclude this section by characterizing the gap to the capacity when H is diagonal and the channel input space is the Cartesian product of *n* PAM constellations. In this context, PAM(N,A) refers to the set of N∈N equidistant PAM-constellation points with amplitude constraint $A\in R_{+}$ (see Figure 3 for an illustration), whereas X∼PAM(N,A) means that *X* is uniformly distributed over PAM(N,A) [11].

**Theorem** **18.** *Let $H=\mathrm{diag}\left(h_{11},\cdots ,h_{nn}\right)\in R^{n\times n}$ be fixed and $X=(X_{1},\cdots ,X_{n})$. Then, if $X_{i}∼\mathrm{PAM}\left(N_{i},A_{i}\right)$, i=1,⋯,n, for some given $a=\left(A_{1},\cdots ,A_{n}\right)\in R_{+}^{n}$, it holds that*

(39) $${\bar{C}}_{\mathrm{Dual},2}\left(\mathrm{Box}\left(a\right),H\right)-{\underline{C}}_{\mathrm{OW}}\left(\mathrm{Box}\left(a\right),H\right)\leq c\cdot n\;bits,$$

*where $N_{i}:=\left\lfloor 1+\frac{2A_{i}\left|h_{ii}\right|}{\sqrt{2\pi e}}\right\rfloor$ and*

$$c:=1+\frac{1}{2}\log_{2}\left(\frac{\pi e}{6}\right)+\frac{1}{2}\log_{2}\left(1+\frac{6}{\pi e}\right)\approx 1.64.$$

*Moreover, if $X_{i}∼\mathrm{PAM}\left(N_{i},A\right)$, i=1,⋯,n, for some given $A\in R_{+}$, it holds that*

(40) $${\bar{C}}_{\mathrm{Dual},2}\left(B_{0}\left(A\right),H\right)-{\underline{C}}_{\mathrm{OW}}\left(B_{0}\left(A\right),H\right)\leq c\cdot n\;bits,$$

*where $N_{i}:=\left\lfloor 1+\frac{2A|h_{ii}|}{\sqrt{n}\sqrt{2\pi e}}\right\rfloor$.*

**Proof.**  Since the channel matrix is diagonal, letting the channel input X be such that its elements $X_{i}$, i=1,⋯,n, are independent, we have that

$$I\left(X;HX+Z\right)=\sum_{i=1}^{n}I\left(X_{i};h_{ii}X_{i}+Z_{i}\right).$$

Let $X_{i}∼\mathrm{PAM}\left(N_{i},A_{i}\right)$ with $N_{i}:=\left\lfloor 1+\frac{2A_{i}\left|h_{ii}\right|}{\sqrt{2\pi e}}\right\rfloor$ and observe that half the Euclidean distance between any pair of adjacent points in $\mathrm{PAM}(N_{i},A_{i})$ is equal to $\Delta_{i}:=A_{i}/\left(N_{i}-1\right)$ (see Figure 3), i=1,⋯,n. To lower bound the mutual information $I(X_{i};h_{ii}X_{i}+Z_{i})$, we use the bound of Theorem 13 for p=2 and $n_{t}=1$. Thus, for some continuous random variable *U* that is uniformly distributed over the interval $\left[-\Delta_{i},\Delta_{i}\right)$ and independent of $X_{i}$, we have that

(41) $$I\left(X_{i};h_{ii}X_{i}+Z_{i}\right)\geq H\left(X_{i}\right)-\frac{1}{2}\log_{2}\left(\frac{\pi e}{6}\right)-\frac{1}{2}\log_{2}\left(\frac{E\left[{\left(U+X_{i}-g\left(Y_{i}\right)\right)}^{2}\right]}{E[U^{2}]}\right).$$

Now, note that the entropy term in Equation (41) can be lower bounded as

(42) $$H\left(X_{i}\right)=\log_{2}\left(\left\lfloor 1+\frac{2A_{i}\left|h_{ii}\right|}{\sqrt{2\pi e}}\right\rfloor \right)\geq \log_{2}\left(1+\frac{2A_{i}\left|h_{ii}\right|}{\sqrt{2\pi e}}\right)+\log_{2}\left(2\right),$$

where we have used that $\left\lfloor x\right\rfloor \geq \frac{x}{2}$ for every x≥1. On the other hand, the last term in Equation (41) can be upper bounded by upper bounding its argument as follows:

(43) $$\begin{array}{cc} \frac{E\left[{\left(U+X_{i}-g\left(Y_{i}\right)\right)}^{2}\right]}{E[U^{2}]} & \overset{a)}{=}1+\frac{3E\left[{\left(X_{i}-g\left(Y_{i}\right)\right)}^{2}\right]}{\Delta_{i}^{2}}\\ & \overset{b)}{\leq }1+\frac{3E\left[Z_{i}^{2}\right]{\left(N_{i}-1\right)}^{2}}{A_{i}^{2}{\left|h_{ii}\right|}^{2}}\\ & =1+\frac{3{\left(N_{i}-1\right)}^{2}}{A_{i}^{2}{\left|h_{ii}\right|}^{2}}\\ & \overset{c)}{\leq }1+\frac{3{\left(\frac{2A_{i}\left|h_{ii}\right|}{\sqrt{2\pi e}}\right)}^{2}}{A_{i}^{2}{\left|h_{ii}\right|}^{2}}\\ & =1+\frac{6}{\pi e}. \end{array}$$

where (a) follows from using that $X_{i}$ and *U* are independent and $E\left[U^{2}\right]=\frac{\Delta_{i}^{2}}{3}$; (b) from using the estimator $g\left(Y_{i}\right)=\frac{1}{h_{ii}}Y_{i}$; and (c) from $N_{i}=\left\lfloor 1+\frac{2A_{i}\left|h_{ii}\right|}{\sqrt{2\pi e}}\right\rfloor \leq 1+\frac{2A_{i}\left|h_{ii}\right|}{\sqrt{2\pi e}}$. Combining Equations (41), (42), and (43) results in the gap in (39).

The proof of the capacity gap in Equation (40) follows along similar lines, which concludes the proof. □

We are also able to determine the gap to the capacity for a general invertible channel matrix.

**Theorem** **19.** *For any X and any invertible H*

$$C\left(X,H\right)-{\underline{C}}_{\mathrm{OW}}\left(X,H\right)\leq \log_{2}\left(\pi n\right)+\frac{n}{2}\log_{2}\left(1+4n\left(4+4n\right)\left(\frac{∥H^{-1}{∥}^{2}r_{\max}^{2}\left(HX\right)}{r_{\min}^{2}\left(X\right)}+\frac{n∥H^{-1}{∥}^{2}}{r_{\min}^{2}\left(X\right)}\right)\right).$$

**Proof.**  Let X be uniformly distributed over a set constructed from an *n*-dimensional cubic lattice with the number of points equal to $N=\left\lfloor ∥\tilde{x}{+Z∥}_{2}^{n}\right\rfloor$, where $\tilde{x}\in HX$ is chosen such that $∥\tilde{x}∥=r_{\max}\left(HX\right)$, and scaled such that it is contained in the input space X. Note that the minimum distance between point in X are given by

$$d_{\min}\left(\mathrm{supp}(X)\right):=\frac{r_{\min}\left(X\right)}{N^{\frac{1}{n}}}.$$

Now, we compute the difference between the moment upper bound of Theorem 9 and the Ozarow–Wyner lower bound of Theorem 13:

(44) $$\begin{array}{cc} {\bar{C}}_{M}\left(X,H\right)-{\underline{C}}_{\mathrm{OW}}\left(X,H\right) & \overset{\left(a\right)}{\leq }\log_{2}\left(∥\tilde{x}{+Z∥}_{2}\right)-H\left(X\right)+\mathrm{gap}\\ & =n\log_{2}\left(\;∥\tilde{x}{+Z∥}_{2}\right)-\log_{2}\left(\left\lfloor ∥\tilde{x}+Z{∥}_{2}^{n}\right\rfloor \right)+\mathrm{gap}\\ & \overset{\left(b\right)}{\leq }\log_{2}\left(2\right)+\mathrm{gap}, \end{array}$$

where (a) follows from Theorem 9 by choosing p=2; and (b) by using the bound $\left\lfloor x\right\rfloor \geq \frac{x}{2}$ for x>1. The next step in the proof consists in bounding the gap term, which requires to upper bound the terms in Equations (23) and (24) individually. Towards this end, choose p=2 and let U be a random vector that is uniformly distributed over a ball of radius $d_{\min}\left(X\right)$. Thus, for (23) it follows

$$\begin{array}{cc} G_{1,2}\left(U,X,g\right) & =n\log_{2}\left(\frac{{∥U+X-g\left(Y\right)∥}_{2}}{{∥U∥}_{2}}\right)\\ & \overset{\left(a\right)}{=}n\log_{2}\left(\frac{∥U-H^{-1}{Z∥}_{2}}{{∥U∥}_{2}}\right)\\ & =\frac{n}{2}\log_{2}\left(1+\frac{∥H^{-1}{Z∥}_{2}^{2}}{{∥U∥}_{2}^{2}}\right)\\ & \overset{\left(b\right)}{=}\frac{n}{2}\log_{2}\left(1+\frac{4\left(4+4n\right)∥H^{-1}{Z∥}_{2}^{2}}{d_{\min}^{2}\left(\mathrm{supp}(X)\right)}\right)\\ & \overset{\left(c\right)}{\leq }\frac{n}{2}\log_{2}\left(1+\frac{\left(4+4n\right)∥H^{-1}{Z∥}_{2}^{2}\;{∥\tilde{x}+Z∥}_{2}^{2}}{r_{\min}^{2}\left(X\right)}\right)\\ & \overset{\left(d\right)}{=}\frac{n}{2}\log_{2}\left(1+\frac{\left(4+4n\right)∥H^{-1}{Z∥}_{2}^{2}\;\left(r_{\max}^{2}\left(HX\right)+n\right)}{r_{\min}^{2}\left(X\right)}\right)\\ & \overset{\left(e\right)}{\leq }\frac{n}{2}\log_{2}\left(1+\frac{4\left(4+4n\right)∥H^{-1}{∥}^{2}{∥Z∥}_{2}^{2}\;\left(r_{\max}^{2}\left(HX\right)+n\right)}{r_{\min}^{2}\left(X\right)}\right)\\ & =\frac{n}{2}\log_{2}\left(1+\frac{4\left(4+4n\right)∥H^{-1}{∥}^{2}n\;\left(r_{\max}^{2}\left(HX\right)+n\right)}{r_{\min}^{2}\left(X\right)}\right). \end{array}$$

where (a) follows by choosing $g\left(Y\right)=H^{-1}Y$: (b) by using ${∥U∥}_{2}^{2}=\frac{r^{2}}{4+2n}$, where $r=\frac{d_{\min}\left(\mathrm{supp}(X)\right)}{2}$ is the radius of an *n*-dimensional ball; (c) from dropping the floor function in the expression for the minimum distance, i.e.,

$$d_{\min}^{-1}\left(\mathrm{supp}(X)\right)=\frac{N^{\frac{1}{n}}}{r_{\min}\left(X\right)}=\frac{{\left\lfloor ∥\tilde{x}{+Z∥}_{2}^{n}\right\rfloor }^{\frac{1}{n}}}{r_{\min}\left(X\right)}\leq \frac{∥\tilde{x}{+Z∥}_{2}}{r_{\min}\left(X\right)};$$

(d) follows by expanding $∥\tilde{x}{+Z∥}_{2}^{2}$ using that $∥\tilde{x}∥=r_{\max}\left(HX\right)$; and (e) from using the bound $∥H^{-1}{Z∥}_{2}\leq ∥H^{-1}{∥∥Z∥}_{2}$.

On the other hand, the term $G_{2,p}\left(U\right)$ can be bounded from above as follows ([36] Appendix L):

$$G_{2,p}\left(U\right)=n\log_{2}\left(\frac{k_{n,p}\;n^{\frac{1}{p}}\;{∥U∥}_{p}}{2^{\frac{1}{n}h\left(U\right)}}\right)\leq n\log_{2}\left({\left(\pi n\right)}^{\frac{1}{n}}\right).$$

Combining these two bounds with the one in (44) provides the result. □

## 6. Arbitrary Channel Matrices

For an arbitrary MIMO channel with an average power constraint, it is well known that the capacity is achieved by a singular value decomposition (SVD) of the channel matrix (i.e., $H=U\Lambda V^{T}$) along with considering the equivalent channel model

$$\tilde{Y}=\Lambda \tilde{X}+\tilde{Z},$$

where $\tilde{Y}:=U^{T}Y$, $\tilde{X}:=V^{T}X$, and $\tilde{Z}:=U^{T}Z$, respectively.

To provide lower bounds for channels with amplitude constraints and SVD precoding, we need the following lemma.

**Lemma** **3.** *For any given orthogonal matrix $V\in R^{n_{t}\times n_{t}}$ and constraint vector $a=\left(A_{1},\cdots ,A_{n_{t}}\right)\in R_{+}^{n_{t}}$, there exists a distribution $F_{X}$ of X such that $\tilde{X}=V^{T}X$ is uniformly distributed over Box(a). Moreover, the components ${\tilde{X}}_{1},\cdots ,{\tilde{X}}_{n_{t}}$ of $\tilde{X}$ are mutually independent with ${\tilde{X}}_{i}$ uniformly distributed over $\left[-A_{i},A_{i}\right]$, $i=1,\cdots ,n_{t}$.*

**Proof.**  Suppose that $\tilde{X}$ is uniformly distributed over Box(a); that is, the density of $\tilde{X}$ is of the form

$$f_{\tilde{X}}\left(\tilde{x}\right)=\frac{1}{\mathrm{Vol}\left(\mathrm{Box}(a)\right)},\;\tilde{x}\in \mathrm{Box}\left(a\right).$$

Since V is orthogonal, we have $V\tilde{X}=X$ and by the change of variable Theorem for x∈VBox(a)

$$\begin{array}{c} f_{X}\left(x\right)=\frac{1}{\left|\det(V)\right|}f_{\tilde{X}}\left(V^{T}x\right)=\frac{1}{|\det\left(V\right)|\mathrm{Vol}\left(\mathrm{Box}(a)\right)}=\frac{1}{\mathrm{Vol}\left(\mathrm{Box}(a)\right)}. \end{array}$$

Therefore, such a distribution of X exists. □

**Theorem** **20.** (Lower Bounds with SVD Precoding) *Let $H\in R^{n_{r}\times n_{t}}$ be fixed, $n_{\min}:=\min\left(n_{r},n_{t}\right)$, and X=Box(a) for some $a=\left(A_{1},\cdots ,A_{n_{t}}\right)\in R_{+}^{n_{t}}$. Furthermore, let $\sigma_{i}$, $i=1,\cdots ,n_{\min}$, be the ith singular value of H. Then,*

(45) $${\underline{C}}_{\mathrm{Jensen}}\left(\mathrm{Box}\left(a\right),H\right)=\log_{2}^{+}\left({\left(\frac{2}{e}\right)}^{\frac{n_{\min}}{2}}\frac{1}{\psi (H,b^{⋆})}\right)$$

*and*

(46) $${\underline{C}}_{\mathrm{EPI}}\left(\mathrm{Box}\left(a\right),H\right)=\frac{n_{\min}}{2}\log_{2}\left(1+\frac{{\left|\prod_{i=1}^{n_{\min}}A_{i}\sigma_{i}\right|}^{\frac{2}{n_{\min}}}}{2\pi e}\right),$$

*where*

$$\psi \left(H,b^{⋆}\right):=\min_{b\in \mathrm{Box}(a)}\prod_{i=1}^{n_{\min}}\varphi \left(\sigma_{i}B_{i}\right)$$

*with $b:=(B_{1},\cdots ,B_{n_{t}})$ and φ as defined in Equation (32).*

**Proof.**  Performing the SVD, the expected value in Theorem 14 can be written as

$$E\left[e^{-\frac{∥H\left(X-X^{\prime }\right){∥}^{2}}{4}}\right]=E\left[e^{-\frac{∥U\Lambda V^{T}\left(X-X^{\prime }\right){∥}^{2}}{4}}\right]=E\left[e^{-\frac{∥\Lambda V^{T}\left(X-X^{\prime }\right){∥}^{2}}{4}}\right]=E\left[e^{-\frac{∥\Lambda \left(\tilde{X}-{\tilde{X}}^{\prime }\right){∥}^{2}}{4}}\right].$$

By Lemma 3, there exists a distribution $F_{X}$ such that the components of $\tilde{X}$ are independent and uniformly distributed. Since Λ is a diagonal matrix, we can use Theorem 16 to arrive at Equation (45).

Note that by Lemma 3 there exists a distribution on X such that $\tilde{X}$ is uniform over $\mathrm{Box}\left(a\right)\subset R^{n_{t}}$ and $\Lambda \tilde{X}$ is uniform over $\Lambda \mathrm{Box}\left(a\right)\subset R^{n_{\min}}$, respectively. Therefore, by the EPI lower bound given in Equation (20), we obtain

$$\begin{array}{cc} {\underline{C}}_{\mathrm{EPI}}\left(\mathrm{Box}\left(a\right),H\right) & =\frac{n_{\min}}{2}\log_{2}\left(1+\frac{2^{\frac{2}{n_{\min}}h\left(\Lambda \tilde{X}\right)}}{2\pi e}\right)\\ & =\frac{n_{\min}}{2}\log_{2}\left(1+\frac{\mathrm{Vol}{\left(\Lambda \mathrm{Box}(a)\right)}^{\frac{2}{n_{\min}}}}{2\pi e}\right)\\ & =\frac{n_{\min}}{2}\log_{2}\left(1+\frac{{\left(\prod_{i=1}^{n_{\min}}A_{i}\right)}^{\frac{2}{n_{\min}}}{\left|\prod_{i=1}^{n_{\min}}\sigma_{i}\right|}^{\frac{2}{n_{\min}}}}{2\pi e}\right), \end{array}$$

which is exactly the expression in Equation (46). This concludes the proof. □

**Remark** **8.** *Notice that choosing the optimal b for the lower bound in Equation (45) is an* amplitude allocation problem, *which is reminiscent of waterfilling in the average power constraint case. It would be interesting to study whether the bound in Equation (45) is connected to what is called* mercury waterfilling *in [37,38].*

**Figure 4:**
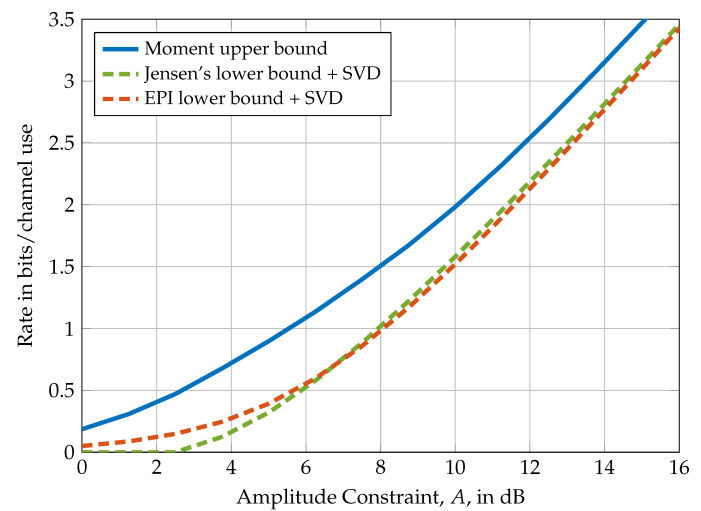
Comparison of the upper bound in Theorem 2 with the lower bounds of Theorem 20 for a 3×1 MIMO system with amplitude constraints $A_{1}=A_{2}=A_{3}=A$ (i.e., a=(A,A,A)) and channel matrix h=(0.6557,0.0357,0.8491).

In Figure 4, the lower bounds of Theorem 20 are compared to the moment upper bound of Theorem 2 for the special case of a 3×1 MIMO channel. Similar to the example presented in Figure 2, the EPI lower bound performs well in the low amplitude regime, while Jensen’s inequality lower bound performs well in the high amplitude regime.

We conclude this section by showing that for an arbitrary channel input space X, in the large amplitude regime the capacity pre-log is given by $\min(n_{r},n_{t})$.

**Theorem** **21.** *Let X be arbitrary and $H\in R^{n_{r}\times n_{t}}$ fixed. Then,*

$$\lim_{r_{\min}\left(X\right)\to \infty }\frac{C(X,H)}{\log_{2}\left(1+\frac{2r_{\min}\left(X\right)}{\sqrt{2\pi e}}\right)}=\min\left(n_{r},n_{t}\right).$$

**Proof.**  Notice that there always exists $a\in R_{+}^{n_{t}}$ and $c\in R_{+}$ such that Box(a)⊆X⊂cBox(a). Thus, without loss of generality, we can consider X=Box(a), a=(A,⋯,A), for sufficiently large $A\in R_{+}$. To prove the result, we therefore start with enlarging the constraint set of the bound in Equation (11):

$$\begin{array}{cc} \mathrm{Box}\left(H\mathrm{Box}(a)\right) & \subseteq B_{0}\left(r_{\max}\left(H\mathrm{Box}(a)\right)\right)\\ & \subseteq B_{0}\left(r_{\max}\left(HB_{0}\left(\sqrt{n_{t}}A\right)\right)\right)\\ & =B_{0}\left(r_{\max}\left(U\Lambda V^{T}B_{0}\left(\sqrt{n_{t}}A\right)\right)\right)\\ & =B_{0}\left(r_{\max}\left(U\Lambda B_{0}\left(\sqrt{n_{t}}A\right)\right)\right)\\ & =B_{0}\left(r_{\max}\left(\Lambda B_{0}\left(\sqrt{n_{t}}A\right)\right)\right)\\ & \subseteq B_{0}\left(r\right)\\ & \subseteq \mathrm{Box}(a^{\prime }), \end{array}$$

where $r:=\sqrt{n_{t}}A\sqrt{\sum_{i=1}^{n_{\min}}\sigma_{i}^{2}}$ and $a^{\prime }:=\left(\frac{r}{\sqrt{n_{\min}}},\cdots ,\frac{r}{\sqrt{n_{\min}}}\right)\in R_{+}^{n_{\min}}$. Therefore, by using the upper bound in Equation (11), it follows that

$$C\left(\mathrm{Box}\left(a\right),H\right)\leq \sum_{i=1}^{n_{r}}\log_{2}\left(1+\frac{2A_{i}}{\sqrt{2\pi e}}\right)\leq n_{\min}\log_{2}\left(1+\frac{2}{\sqrt{2\pi e}}\frac{\sqrt{n_{t}}A\sqrt{\sum_{i=1}^{n_{\min}}\sigma_{i}^{2}}}{\sqrt{n_{\min}}}\right).$$

Moreover,

$$\lim_{A\to \infty }\frac{C(\mathrm{Box}(a),H)}{\log_{2}\left(1+\frac{2A}{\sqrt{2\pi e}}\right)}\leq n_{\min}\lim_{A\to \infty }\frac{\log_{2}\left(1+\frac{2}{\sqrt{2\pi e}}\frac{\sqrt{n_{t}}A\sqrt{\sum_{i=1}^{n_{\min}}\sigma_{i}^{2}}}{\sqrt{n_{\min}}}\right)}{\log_{2}\left(1+\frac{2A}{\sqrt{2\pi e}}\right)}=n_{\min}.$$

Next, using the EPI lower bound in Equation (46), we have that

$$\lim_{A\to \infty }\frac{{\underline{C}}_{\mathrm{EPI}}\left(\mathrm{Box}\left(a\right),\Lambda \right)}{\log_{2}\left(1+\frac{2A}{\sqrt{2\pi e}}\right)}=n_{\min}\lim_{A\to \infty }\frac{\frac{1}{2}\log_{2}(1+\frac{A{\left|\prod_{i=1}^{n_{\min}}\sigma_{i}\right|}^{\frac{2}{n_{\min}}}}{2\pi e})}{\log_{2}\left(1+\frac{2A}{\sqrt{2\pi e}}\right)}=n_{\min}.$$

This concludes the proof. □

## 7. The SISO Case

In this section, we apply the upper and lower bounds presented in the previous sections to the special case of a SISO channel that is subject to an amplitude constraint (i.e., X=[−A,A] for some $A\in R_{+}$) and compare them with the state-of-the art. More precisely, we are interested in upper and lower bounds to the capacity

(47) $$C\left(\left[-A,A\right],h\right):=\max_{F_{X}:X\in \left[-A,A\right]}I\left(X;hX+Y\right).$$

Without loss of generality, we assume h=1 in all that follows.

### 7.1. Upper and Lower Bounds

As a starting point for our comparisons, the following Theorem summarizes bounds on the capacity (47) that are known from the literature. The bounds are all based on the duality approach that we generalize in Section 4 to the MIMO case.

**Theorem** **22.** (Known Duality Upper Bounds) *Let A>0 be arbitrary. Then, the following are valid upper bounds to the capacity of the amplitude-constrained SISO channel defined in Equation (47).*

- *McKellips upper bound [7]:*
  (48) $$C\left(\left[-A,A\right],1\right)\leq {\bar{C}}_{\mathrm{McK}}\left(\left[-A,A\right],1\right):=\log_{2}\left(1+\frac{2A}{\sqrt{2\pi e}}\right).$$
- *Thangaraj–Kramer–Böcherer upper bound ([8] Theorem 1):*
  (49) $$\begin{array}{c} C\left(\left[-A,A\right],1\right)\leq {\bar{C}}_{\mathrm{TKB}}\left(\left[-A,A\right],1\right):=\left\{\begin{array}{cc} \beta \left(A\right)\log_{e}\left(\sqrt{\frac{2}{\pi e}}A\right)+H_{b}\left(\beta (A)\right), & A^{2}\leq 6.304\;\mathrm{dB}\\ {\bar{C}}_{\mathrm{McK}}\left(\left[-A,A\right],1\right), & \mathrm{else}, \end{array}\right. \end{array}$$
  *where $\beta \left(A\right):=\frac{1}{2}-Q\left(2A\right)$ and $H_{b}$ denotes the binary entropy function.*
- *Rassouli–Clerckx upper bound [9]:*
  (50) $$C\left(\left[-A,A\right],1\right)\leq {\bar{C}}_{\mathrm{RC}}\left(\left[-A,A\right],1\right):={\bar{C}}_{\mathrm{TKB}}\left(\left[-A,A\right],1\right)+W\left(A\right),$$
  *where*
  $$W\left(A\right):=\frac{1}{2}\left(\log_{e}\left(\sigma^{2}\left(A\right)\right)+\frac{1}{\sigma^{2}\left(A\right)}-1\right)\left(\frac{1}{2}+Q\left(2A\right)\right)+\frac{g(2A)}{2\sigma^{2}\left(A\right)},$$
  $$\sigma^{2}\left(A\right):=1+\frac{2g(2A)}{1+2Q(2A)},$$
  *and*
  $$g\left(x\right):=x^{2}Q\left(x\right)-\frac{x}{\sqrt{2\pi }}e^{-\frac{x^{2}}{2}}.$$

Now, we apply the moment upper bound of Theorem 9 to the SISO case.

**Theorem** **23.** (Moment Upper Bound) *Let A>0 be arbitrary. Then,*

(51) $$C\left(\left[-A,A\right],1\right)\leq {\bar{C}}_{M}\left(\left[-A,A\right],1\right)=\underset{p>0}{\inf}\log_{2}\left(\frac{k_{1,p}}{{\left(2\pi e\right)}^{\frac{1}{2}}}\;E{\left[{\left|A+Z\right|}^{p}\right]}^{\frac{1}{p}}\right),$$

*where the expected value is of the explicit form*

$$E\left[{\left|A+Z\right|}^{p}\right]=\frac{2^{\frac{p}{2}}\Gamma \left(\frac{p+1}{2}\right)}{\sqrt{\pi }}\;{}_{1}F_{1}\left(-\frac{p}{2};\frac{1}{2};-\frac{A^{2}}{2}\right)$$

*with ${}_{1}F_{1}\left(a;b;z\right)$ being the confluent hypergeometric function of the first kind ([39] Chapter 13).*

**Proof.**  First, note that $r_{\max}\left(\left[-A,A\right]\right)=A$. Then, by using the expression for the raw absolute moment of a Gaussian distribution given in [40], we have that

$$\max_{a\in [0,A]}E\left[{\left|a+Z\right|}^{p}\right]=\max_{a\in [0,A]}\frac{2^{\frac{p}{2}}\Gamma \left(\frac{p+1}{2}\right)}{\sqrt{\pi }}\;{}_{1}F_{1}\left(-\frac{p}{2};\frac{1}{2};-\frac{a^{2}}{2}\right).$$

The proof is concluded by observing that $f\left(a\right):={}_{1}F_{1}\left(-\frac{p}{2};\frac{1}{2};-\frac{a^{2}}{2}\right)$ is an increasing function in *a*. □

The following theorem establishes the EPI and the Jensen lower bound of Section 4.2 assuming the channel input symbols are uniformly distributed.

**Theorem** **24.** (Lower Bounds with Uniform Inputs) *Let A>0 be arbitrary and the channel input X be uniformly distributed over [−A,A]. Then,*

(52) $$C\left(\left[-A,A\right],1\right)\geq {\underline{C}}_{\mathrm{EPI}}\left(\left[-A,A\right],1\right)=\frac{1}{2}\log_{2}\left(1+\frac{2A^{2}}{\pi e}\right)$$

*and*

(53) $$C\left(\left[-A,A\right],1\right)\geq {\underline{C}}_{\mathrm{Jensen}}\left(\left[-A,A\right],1\right)=\log_{2}\left(\frac{\sqrt{2}A^{2}}{e^{\frac{1}{2}}\left(e^{-A^{2}}-1+\sqrt{\pi }A\left(1-2Q\left(\sqrt{2}A\right)\right)\right)}\right).$$

**Proof.**  The lower bound in Equation (52) follows from Theorem 12 by observing that Vol(X)=2A. To show the lower bound in Equation (53), consider Theorem 14 and let *X* and $X^{\prime }$ be independent and uniformly distributed over [−A,A]. Then, we have

$$\begin{array}{cc} E\left[e^{-\frac{|X-X^{\prime }{|}^{2}}{4}}\right] & =\frac{1}{4A^{2}}\int_{-A}^{A}\int_{-A}^{A}e^{-\frac{{\left(x-x^{\prime }\right)}^{2}}{4}}dx\;dx^{\prime }\\ & =\frac{1}{A^{2}}\left(e^{-A^{2}}-1+\sqrt{\pi }A\left(1-2Q\left(\sqrt{2}A\right)\right)\right), \end{array}$$

which concludes the proof. □

Restricting the channel inputs to be discrete allows for another set of lower bounds on Equation (47).

**Theorem** **25.** (Lower Bounds with Discrete Inputs) *Let A>1 be arbitrary, $X_{B}\in \left\{-A,A\right\}$ equally likely, and $X_{D}∼\mathrm{PAM}\left(N\right)$ with $N=\left\lceil 1+\frac{A}{\sqrt{2\pi e}}\right\rceil$. Then,*

(54) $$\begin{array}{cc} C\left(\left[-A,A\right],1\right)\geq {\underline{C}}_{\mathrm{Binary}}\left(\left[-A,A\right],1\right) & :=I(X_{B};X_{B}+Z) \end{array}$$

(55) $$\begin{array}{c} =\frac{1}{\log_{e}\left(2\right)}\left(A^{2}-\int_{-\infty }^{\infty }\frac{e^{-\frac{y^{2}}{2}}}{\sqrt{2\pi }}\log_{e}\left(\cosh(A^{2}-Ay)\right)dy\right), \end{array}$$

(56) $$C\left(\left[-A,A\right],1\right)\geq {\underline{C}}_{\mathrm{Jensen}}\left(\left[-A,A\right],1\right)=-\log_{2}\left(\sqrt{\frac{e}{2}}\frac{1}{N^{2}}\sum_{\left(x_{D_{i}},x_{D_{j}}\right)\in \mathrm{PAM}{\left(N\right)}^{2}}e^{-\frac{{\left(x_{D_{i}}-x_{D_{j}}\right)}^{2}}{4}}\right),$$

*and*

(57) $$C\left(\left[-A,A\right],1\right)\geq {\underline{C}}_{\mathrm{OW}}\left(\left[-A,A\right],1\right)={\bar{C}}_{\mathrm{McK}}\left(\left[-A,A\right],1\right)-2.$$

**Proof.**  The expression of the mutual information in Equation (54) for a uniform binary input $X_{B}\in \left\{-A,A\right\}$ is found in [41] by using the I-MMSE relationship. The bound in Equation (56) follows from using Theorem 14 and the bound in Equation (57) from Theorem 13, respectively. This concludes the proof. □

**Figure 5:**
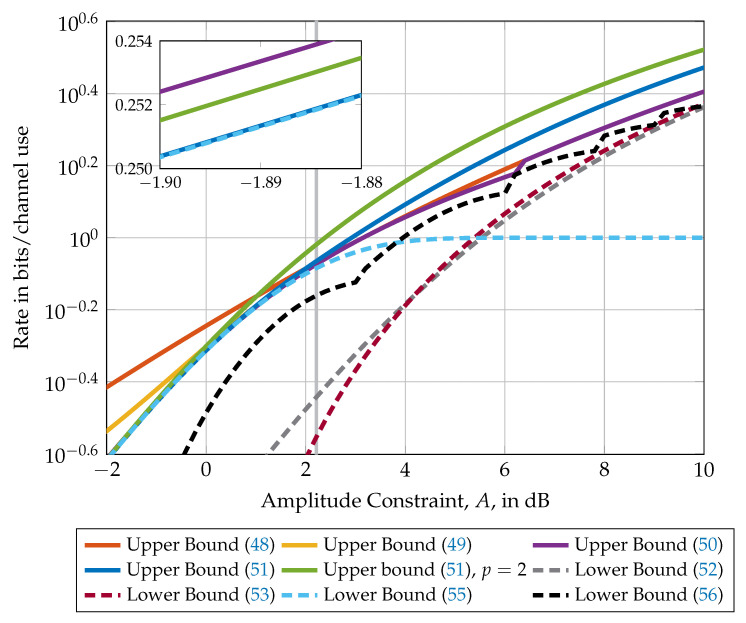
Comparison of upper and lower bounds on the capacity of a SISO channel with amplitude constraint *A*. The capacity of this channel is known for amplitudes smaller than $A\approx 10\log_{10}\left(1.665\right)=2.214\;\mathrm{dB}$ only (i.e., to the left of the gray vertical line) and unknown elsewhere. The nested figure represents a zoom into the region −1.9dB≤A≤−1.88dB to highlight the differences between the Moment upper bound (51), the Rassouli–Clerckx upper bound in Equation (50), and the lower bound with binary inputs in Equation (56).

Figure 5 compares the upper and lower bounds presented in this section in dependency of the amplitude constraint *A*. Observe that for values of *A* smaller than ≈1.665 (i.e., to the left of the gray vertical line), the lower bound (55) is in fact equal to the capacity. Up to constraints of A≈1, the moment upper bound in Equation (51) is the best after which the bound in Equation (50) becomes the tightest. The best lower bound for constraint values smaller than A≈10 is the bound in Equation (56) after which the lower bound in Equation (53) becomes the tightest. Note that all lower and upper bounds are asymptotically tight (i.e., for A→∞).

### 7.2. High and Low Amplitude Asymptotics

In this subsection, we study how the capacity in Equation (47) behaves in the high and low amplitude regimes. To this end, we need the following expression

$${\bar{C}}_{\mathrm{AWGN}}\left(\left[-A,A\right],1\right):=\frac{1}{2}\log_{2}\left(1+A^{2}\right),$$

which is either the capacity of a SISO channel with an average power constraint $A^{2}$ or the moment bound in Equation (51) evaluated for p=2.

**Theorem** **26.** (SISO High and Low Amplitude Asymptotics) *It holds*

(58) $$\lim_{A\to 0}\frac{C([-A,A],1)}{{\bar{C}}_{\mathrm{AWGN}}\left(\left[-A,A\right],1\right)}=1,$$

(59) $$\lim_{A\to \infty }\frac{C([-A,A],1)}{{\bar{C}}_{\mathrm{McK}}\left(\left[-A,A\right],1\right)}=1,$$

*and*

(60) $$\lim_{A\to \infty }C\left(\left[-A,A\right],1\right)-{\bar{C}}_{\mathrm{AWGN}}\left(\left[-A,A\right],1\right)=\frac{1}{2}\log_{2}\left(\frac{\pi e}{2}\right)\approx 1.044.$$

**Proof.**  The capacity of an amplitude-constrained SISO channel in the regime of low amplitudes (i.e., for amplitudes smaller than A≈1.655) was given by Guo et al. [41]

$$C\left(\left[-A,A\right],1\right)=\frac{1}{\log_{e}\left(2\right)}\left(A^{2}-\int_{-\infty }^{\infty }\frac{e^{-\frac{y^{2}}{2}}}{\sqrt{2\pi }}\log_{e}\left(\cosh(A^{2}-Ay)\right)dy\right).$$

Now, observe that

$$\begin{array}{c} \lim_{A\to 0}\frac{1}{\frac{1}{2}\log_{2}\left(1+A^{2}\right)}\int_{-\infty }^{\infty }\frac{e^{-\frac{y^{2}}{2}}}{\sqrt{2\pi }}\log_{e}\left(\cosh(A^{2}-Ay)\right)dy\\ =\frac{2}{\log_{2}\left(e\right)}\int_{-\infty }^{\infty }\frac{e^{-\frac{y^{2}}{2}}}{\sqrt{2\pi }}\frac{\log_{e}\left(\cosh(A^{2}-Ay)\right)}{\log_{e}\left(1+A^{2}\right)}\;dy\\ =\frac{2}{\log_{2}\left(e\right)}\int_{-\infty }^{\infty }\frac{e^{-\frac{y^{2}}{2}}}{\sqrt{2\pi }}\lim_{A\to 0}\frac{\log_{e}\left(\cosh(A^{2}-Ay)\right)}{\log_{e}\left(1+A^{2}\right)}\;dy\\ =\frac{2}{\log_{2}\left(e\right)}\int_{-\infty }^{\infty }\frac{e^{-\frac{y^{2}}{2}}}{\sqrt{2\pi }}\frac{y^{2}}{2}dy\\ =\frac{1}{\log_{2}\left(e\right)}. \end{array}$$

Therefore, the limit in Equation (58) is given by

$$\begin{array}{cc} \lim_{A\to 0}\frac{C([-A,A],1)}{{\bar{C}}_{\mathrm{AWGN}}\left(\left[-A,A\right],1\right)} & =\lim_{A\to 0}\frac{A^{2}}{\log_{e}\left(2\right)\frac{1}{2}\log_{2}\left(1+A^{2}\right)}-\frac{1}{\log_{e}\left(2\right)\log_{2}\left(e\right)}\\ & =\frac{2\log_{e}\left(2\right)}{\log_{e}\left(2\right)}-\frac{1}{\log_{e}\left(2\right)\log_{2}\left(e\right)}\\ & =2-1=1. \end{array}$$

The limit in Equation (59) follows from comparing the EPI lower bound ${\underline{C}}_{\mathrm{EPI}}\left(\left[-A,A\right],1\right)=\frac{1}{2}\log_{2}\left(1+\frac{2A^{2}}{\pi e}\right)$ in (52) with the McKellips upper bound ${\bar{C}}_{\mathrm{McK}}\left(\left[-A,A\right],1\right)=\log_{2}\left(1+\frac{2A}{\sqrt{2\pi e}}\right)$ given in Equation (48).

Finally, to show Equation (60), observe that

$$\begin{array}{cc} \lim_{A\to \infty }C\left(\left[-A,A\right],1\right)-{\bar{C}}_{\mathrm{AWGN}}\left(\left[-A,A\right],1\right) & =\lim_{A\to \infty }{\bar{C}}_{\mathrm{McK}}\left(\left[-A,A\right],1\right)-{\bar{C}}_{\mathrm{AWGN}}\left(\left[-A,A\right],1\right)\\ & =\lim_{A\to \infty }\log_{2}\left(1+\frac{2A}{\sqrt{2\pi e}}\right)-\frac{1}{2}\log_{2}\left(1+A^{2}\right)\\ & =\frac{1}{2}\log_{2}\left(\frac{\pi e}{2}\right). \end{array}$$

This concludes the proof. □

## 8. Conclusions

In this work, we studied the capacity of MIMO channels with bounded input spaces. Several new properties of input distributions that achieve the capacity of such channels have been provided. In particular, it is shown that the support of a capacity-achieving channel input distribution is a set that is small in a topological and measure theoretical sense. In addition to that, it is shown that, if the radius of the underlying channel input space, X, is small enough, then the support of a corresponding capacity-achieving input distribution must necessarily be a subset of the boundary of X. As the considerations on the input distribution have demonstrated that determining the capacity is a very challenging problem, we proposed several new upper and lower bounds that are shown to be tight in the high amplitude regime. An interesting future direction would be to study generalizations of our techniques to wireless optical MIMO channels [42] and other channels such as the wiretap channel [43].
